# High Density 3D Carbon Tube Nanoarray Electrode Boosting the Capacitance of Filter Capacitor

**DOI:** 10.1007/s40820-024-01458-6

**Published:** 2024-07-03

**Authors:** Gan Chen, Fangming Han, Huachun Ma, Pei Li, Ziyan Zhou, Pengxiang Wang, Xiaoyan Li, Guowen Meng, Bingqing Wei

**Affiliations:** 1 Key Laboratory of Materials Physics, and Anhui Key Laboratory of Nanomaterials and Nanotechnology, Institute of Solid State Physics, HFIPS, Chinese Academy of Sciences, Hefei, 230031 People’s Republic of China; 2 Department of Materials Science and Engineering, University of Science and Technology of China, Hefei, 230026 People’s Republic of China; 3 Department of Mechanical Engineering, University of Delaware, Newark, DE 19716 USA; 4 Mechano-X Institute, Applied Mechanics Laboratory, Department of Engineering Mechanics, Tsinghua University, Beijing, 100084 People’s Republic of China

**Keywords:** Compactly arranged, Three-dimensional carbon tube nanoarray, Dimensional carbon tube nanoarray, Fast frequency response, Electric double-layer capacitors, Layer capacitors, AC line-filtering, Filtering

## Abstract

Electric double-layer capacitors (EDLCs) with fast frequency response are regarded as small-scale alternatives to the commercial bulky aluminum electrolytic capacitors. Creating carbon-based nanoarray electrodes with precise alignment and smooth ion channels is crucial for enhancing EDLCs’ performance. However, controlling the density of macropore-dominated nanoarray electrodes poses challenges in boosting the capacitance of line-filtering EDLCs. Herein, a simple technique to finely adjust the vertical-pore diameter and inter-spacing in three-dimensional nanoporous anodic aluminum oxide (3D-AAO) template is achieved, and 3D compactly arranged carbon tube (3D-CACT) nanoarrays are created as electrodes for symmetrical EDLCs using nanoporous 3D-AAO template-assisted chemical vapor deposition of carbon. The 3D-CACT electrodes demonstrate a high surface area of 253.0 m^2^ g^−1^, a D/G band intensity ratio of 0.94, and a C/O atomic ratio of 8. As a result, the high-density 3D-CT nanoarray-based sandwich-type EDLCs demonstrate a record high specific areal capacitance of 3.23 mF cm^−2^ at 120 Hz and exceptional fast frequency response due to the vertically aligned and highly ordered nanoarray of closely packed CT units. The 3D-CT nanoarray electrode-based EDLCs could serve as line filters in integrated circuits, aiding power system miniaturization.

**Supplementary Information:**

The online version contains supplementary material available at 10.1007/s40820-024-01458-6.

## Highlights

- A novel method is developed for precise control over the structure of 3D anodic aluminum oxide templates, enabling fine-tuning of both the vertical pore diameter and interspace within the templates.
- 3D carbon tube nanoarrays featuring significantly thinner and denser tubes are constructed as high-quality electrodes for miniaturized filter capacitors.
- The 3D compactly arranged carbon tube-based capacitor achieves a remarkable specific areal capacitance of 3.23 mF cm^−2^ with a phase angle of − 80.2° at 120 Hz.

**Supplementary Information:**

The online version contains supplementary material available at 10.1007/s40820-024-01458-6.

## Graphical abstract

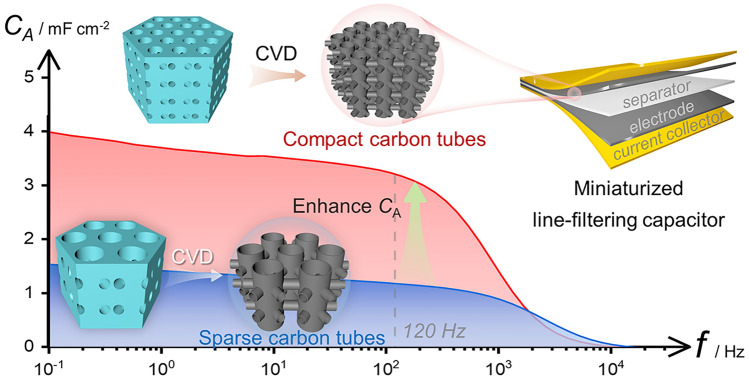

## Introduction

The boom in portable and wearable electronic devices calls for highly integrated circuits and miniaturized components [1–6]. Alternating current (AC)/direct current (DC) conversion is fundamental for powering electronic products [7–9]. Filter capacitors are utilized to smooth the pulse DC voltage after rectification [10–12]. Conventional aluminum electrolytic capacitors (AECs), with bulky size and rigid attributes, dominate the market in the line-filtering field and face the challenges of integrating into miniaturized devices [13–16]. Fast frequency-responsive electric double-layer capacitors (EDLCs) are promising candidates for line-filtering AECs and have attracted extensive attention [17–20]. However, achieving tiny EDLCs with both high energy density and high-frequency response properties remains challenging. The search for high-quality electrode materials with large specific surface areas and efficient electron transport and ion migration paths is critical to developing miniaturized line-filtering EDLCs.

Various novel carbon-based electrode materials for line-filtering EDLCs have been explored [21–28]. Of particular interest are the three-dimensional (3D) upright nanoarrays because of the orientation of the electrode microstructures, which provide smooth ion migration channels [29–33]. However, the fast frequency response of these macropore-dominated electrodes is maintained by sacrificing the energy density, resulting in a low specific areal capacitance (*C*_A_) at 120 Hz, hindering the development of miniaturized line-filtering capacitors [34–36]. Moreover, it is challenging to control the arrangement density of upright nanoarrays using existing methods [22, 37–39]. Therefore, developing a proper way to manage the arrangement of high-orientation nanoarrays to enhance *C*_A_ is of great significance.

The nanoporous anodic aluminum oxide (AAO) template-assisted method is suitable for preparing ordered, highly oriented, and uniform nanoarrays with controllable morphology and high repeatability [40–42]. We have constructed 3D carbon tube (3D-CT) grid electrodes fabricated with the help of a unique nanoporous 3D-AAO template-assisted method for high-performance line-filtering EDLCs [8]. The 3D-CTs grid, i.e., a large area of highly-ordered and vertically aligned CTs with the nearest neighboring vertical CTs being interconnected by lateral CTs growing from vertical CTs, holds great potential for fast frequency response and ultrahigh-power energy storage devices [43–46]. Unfortunately, the sizeable vertical pore diameter (*D*_P_, ~ 250 nm) and interpore distance (*D*_int_, ~ 450 nm) of the phosphoric acid-anodized 3D-AAO template limit the specific surface area of the resultant 3D-CT grids and, consequently, *C*_A_ of the fabricated line-filtering EDLCs. We recently explored 3D multi-layer CT electrodes with a “Russian matryoshka doll” design to increase the specific surface area and *C*_A_ for these applications [46]. While these EDLCs achieved significantly enhanced *C*_A_ by incorporating smaller coaxial CTs within the larger ones, the fabrication process was very complicated and required stringent control. A more practical approach would be desirable to enhance the vertical-pore density of the 3D-AAO templates directly. This would enable to create 3D-CT arrays with a high packing density of smaller-diameter verical CTs in a simple 3D-AAO template assisted chemical vapor deposition (CVD) of carbon, leading to a substantial increase in *C*_A_. Up to now, porous 3D-AAO templates have been only achieved by anodizing Al foil with trace impurity in phosphoric acid electrolyte under about 195 V voltage, and with very few specific *D*_P_ and *D*_int_ have been documented [47, 48]. Therefore, it remains challenging to achieve near-continuous control of the vertical pore with small diameter in the nanoporous 3D-AAO templates, and a more in-depth exploration to regulate vertical pore with small diameter in 3D-AAO templates is necessary to increase the density of the resultant vertically aligned 3D-CT arrays and thus enhance the *C*_A_ of the 3D-CT nanoarray electrode based line-filtering EDLCs.

Herein, we developed a simple and efficient method to systematically reduce the vertical-pore diameter of the 3D-AAO template by adjusting the anodization conditions, including voltage, electrolyte composition, and temperature. Consequently, compared with those of the phosphoric acid anodized 3D-AAO template under 195 V, the vertical pore diameter *D*_P_ and interpore distance *D*_int_ of the new 3D-AAO template can be continuously adjustable to smaller ones of 70–250 and 110–450 nm, respectively. We used these new 3D-AAOs with smaller *D*_P_ and *D*_int_ as templates to prepare 3D compactly arranged CT (denoted as 3D-CACT) nanoarrays via the CVD method. The ordered 3D-CACT nanoarrays with tightly packed CT units provide rich and accessible specific surface area and rapid ion transport paths, making it a high-quality electrode for line-filtering EDLCs, resulting in a high *C*_A_ of 3.23 mF cm^−2^ and a phase angle of -80.2° at 120 Hz, and exhibiting great potential for compact line-filtering applications. The 3D-CACT-based EDLCs can function as filter capacitors in integrated circuits, contributing to the miniaturization of power transmission systems.

## Experimental Section

### Materials

Commercial Al foils with a purity of 99% and a thickness of 0.1 mm were purchased from Shanghai Dingxin Materials Co., Ltd. (China). Other chemicals and reagents, including concentrated phosphoric acid (H_3_PO_4_), oxalic acid (H_2_C_2_O_2_), sulphuric acid (H_2_SO_4_), tin tetrachloride (SnCl_4_), hydrofluoric acid (HF), were supplied from Sinopharm Chemical Reagent Co., Ltd. (China).

### Preparations of 3D-AAOs and 3D-CTs

#### Preparations of the 3D-AAO Templates

Using 0.3 M phosphoric acid (H_3_PO_4_) solution as the electrolyte, Al foil with Cu impurities (99% purity, 100 μm thick) as the positive electrode, graphite as the negative electrode, the anodic oxidation was carried out at 0 °C and a DC constant voltage of 195 V. The anodizing duration could control the thicknesses of 3D-AAO templates. The remaining aluminum (that had not been anodized) was removed in a saturated tin tetrachloride (SnCl_4_) solution, then, the sample was immersed in a 5 wt% H_3_PO_4_ solution at 40 °C for 15 min to obtain the 3D-AAO templates with large vertical pore diameter* D*_P_ and interpore distance *D*_int_. To achieve reduced vertical pore diameter *D*_P_ and interpore distance *D*_int_ in 3D-AAO templates, a combination of lower anodization voltage and a suitable electrolyte is necessary. The smallest *D*_P_ and *D*_int_ were obtained using a 0.3 M oxalic acid (H_2_C_2_O_4_) solution at 10 °C and an anodization voltage of 50 V. For a broader range of *D*_P_ and *D*_int_ values, a mixed electrolyte (0.1 M H_2_C_2_O_4_ + 0.05 M H_3_PO_4_) at 10 °C was employed with anodization voltages of 55, 65, 85, 105, 125, 140, and 155 V.

#### Preparations of the 3D-CT Nanoarrays

The as-prepared 3D-AAO templates were first placed into a horizontal furnace and heated to 1000 °C at 10 °C min^−1^ under an Ar atmosphere. Then, the carbon tubes were grown on the pore walls of the template by pyrolyzing acetylene at 1000 °C, with a flow gas C_2_H_2_ at 60 standard cubic centimeters per minute (sccm) under the pressure of − 0.1 MPa. The resultant samples were then cleaned by Ar plasma with a Plasma Cleaner (Harrick Plasma, PDC-32G) for 30 min to remove the surface amorphous carbon layer and then immersed in 40 wt% hydrofluoric acid solution for 48 h to selectively remove the AAO templates. Finally, after several rinses in deionized water, the 3D-CT nanoarrays were achieved.

### Assembly of EDLCs

For the aqueous system, the symmetrical EDLC was assembled using two identical 3D-CT nanoarrays (the area of each piece is about 0.09 cm^2^) as electrodes, platinum sheets as current collectors, 1 M H_2_SO_4_ as the electrolyte, and the NKK-MPF30AC-100 film as the separator. For the organic system, the electrolyte and the separator were replaced by 1 M tetraethylammonium tetrafluoroborate (TEA-BF_4_, Sigma-Aldrich) dissolving in dry acetonitrile (Sigma-Aldrich) and NKK-TF4030, respectively. Subsequently, the assembled prototype devices were packaged with polyethylene terephthalate (PET) films.

## Results and Discussion

### Design Strategy and Structural Characterizations

**Fig. 1:**
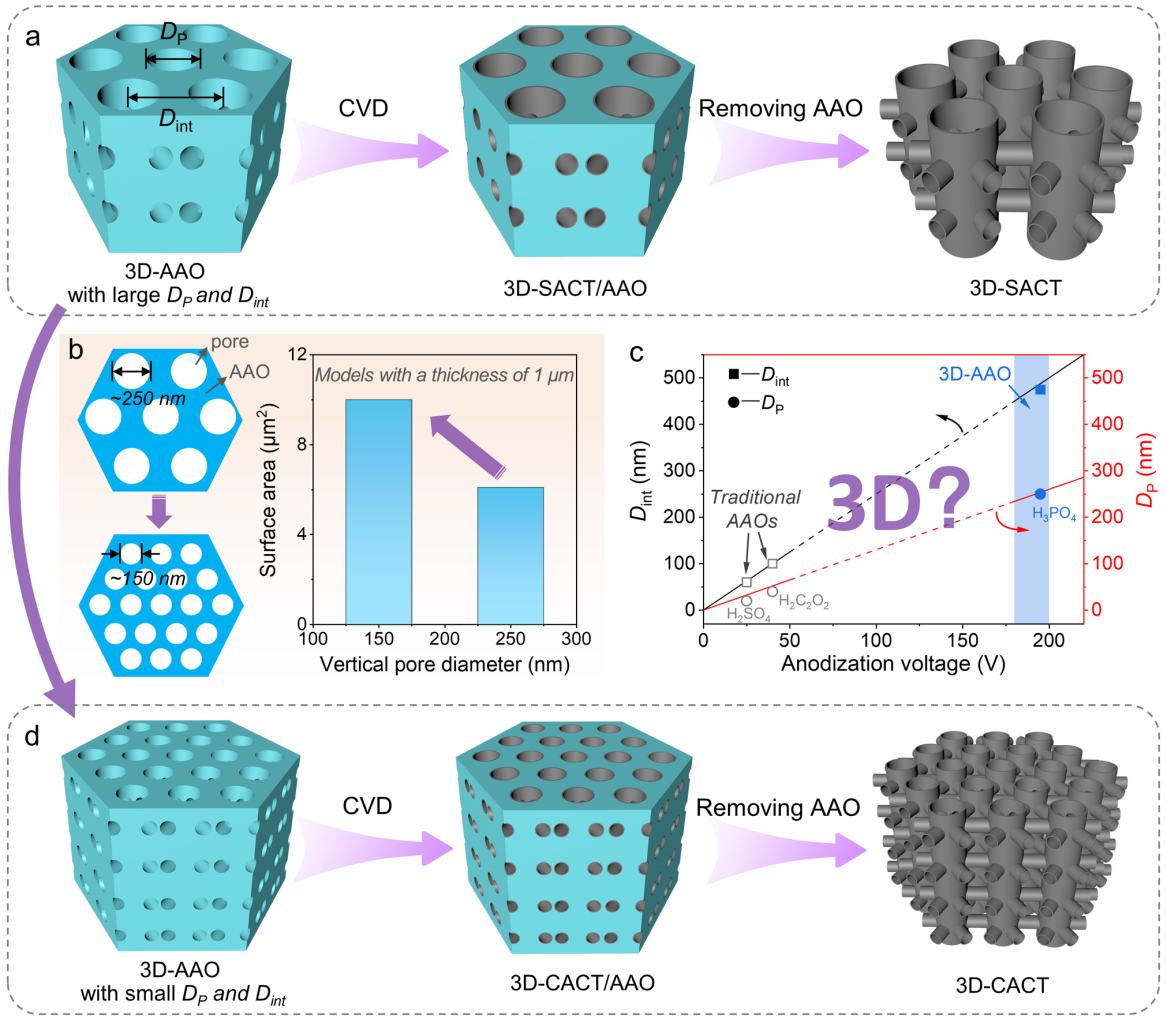
Schematic illustration of concepts and synthesis processes for 3D-SACT and 3D-CACT. **a** Preparation of 3D-SACT using a 3D-AAO template with large *D*_P_ and *D*_int_. **b** Plots of pore surface area for two ideal AAO models vs. vertical pore diameter. **c** Plots of vertical *D*_P_ and* D*_int_ of the AAO vs. anodization voltage. **d** Preparation of 3D-CACT using a 3D-AAO template with small *D*_P_ and *D*_int_

The concept of regulating the vertical pore diameter *D*_P_ and interpore distance *D*_int_ of 3D-AAO templates to increase the CT density of the resultant CVD 3D-CTs nanoarray is illustrated in Fig. 1. The conventional method for preparing 3D-AAO template involves anodizing aluminum foil containing trace impurities with a 0.3 M phosphoric acid solution as an electrolyte at 0 °C under a high voltage of 195 V. The resulting porous 3D-AAO with large vertical pore diameter *D*_P_ (~ 250 nm) and large vertical-pore interspacing *D*_int_ (~ 450 nm) is used as a template, followed by CVD to obtain a 3D Sparsely Arranged CT (denoted as 3D-SACT) nanoarray after removing the AAO (Fig. 1a). However, this macropore-dominated 3D-CT nanoarray exhibits inefficient space utilization, resulting in a lower specific surface area. By simultaneously reducing both the vertical pore diameter* D*_P_ and interpore distance *D*_int_, the pore surface area of the AAO template can be effectively enhanced (Fig. 1b). This, in turn, leads to a substantial increase in the specific surface area of the resultant CT nanoarray. Traditionally, achieving this requires lowering the anodization voltage based on the well-established linear relationship between pore structure and voltage observed in traditional AAO templates. However, existing methods for preparing traditional straight-pore AAO templates at low voltages (< 185 V, Fig. 1c) are not directly applicable to fabricating 3D-AAO templates with lateral pores. This limitation presents a challenge in obtaining 3D-AAO templates with both small *D*_P_ and *D*_int_. This ability is critical for producing the 3D-CACT with high CT density and specific surface area (Fig. 1d).

This goal is achieved by rational controlling anodization parameters such as voltage, electrolyte composition, and temperature. Traditionally, low voltage anodization leads to small *D*_P_ and *D*_int_, and the acidic electrolyte should be replaced to match the voltage window, for example, usually 25 V anodization for 0.3 M sulphuric acid, 40 V anodization for 0.3 M oxalic acid, and 195 V anodization for 0.3 M phosphoric acid. Herein, we used Al foil with trace Cu impurity as the starting material to prepare the 3D-AAO template and utilized 0.3 M sulphuric acid as the electrolyte and anodized at 0 °C under 25 and 30 V, respectively. The experiment results show that there are almost no lateral pores under the voltages lower than 30 V (Fig. S1), which could be ascribed to the fact that a relatively high voltage is required to drive Cu impurities accumulation in the AAO pore wall during the anodization [47]. Then, we used 0.3 M oxalic acid as the electrolyte and anodized at 10 °C under 40, 45, and 50 V, respectively. It was observed that when the voltage was lower than 50 V, fewer lateral pores were formed (Fig. S2), reaffirming that a high voltage is needed to anodize Al foil with trace Cu impurity to form lateral pores in the preparation of the 3D-AAO template. Therefore, when oxalic acid is used, the 3D-AAO templates should be prepared under a high voltage of over 50 V. Subsequently, a mixed electrolyte of oxalic acid and phosphoric acid is used to bridge the voltage gap between 50 and 195 V. As a result, 3D-AAO templates with smaller vertical pore diameter *D*_P_ and interpore distance *D*_int_ were successfully prepared at voltages of 50–155 V. Using these new 3D-AAO templates, 3D-CTs with different vertical tube diameters and inter-tube spacings were fabricated via CVD processes.

**Fig. 2:**
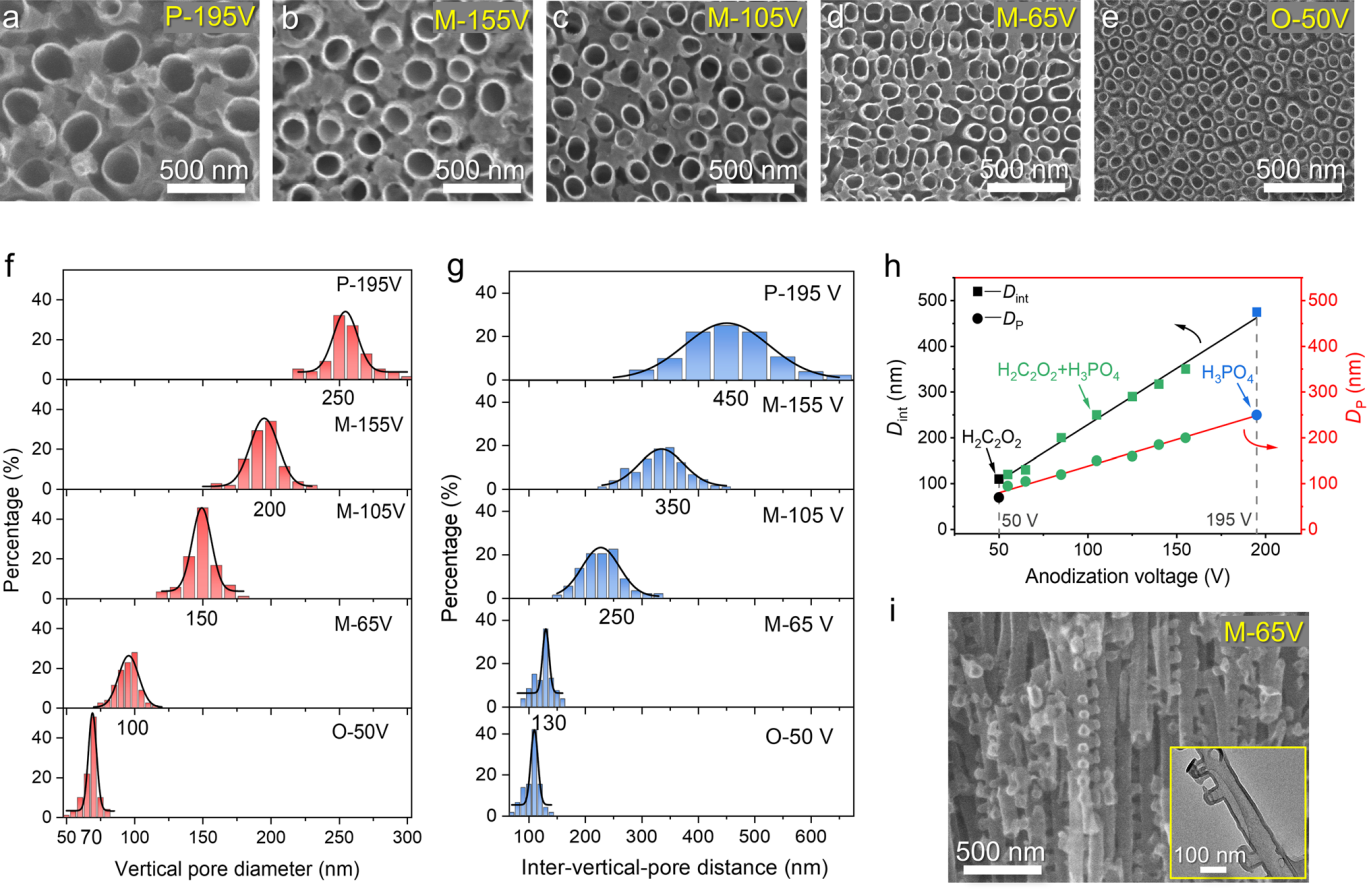
Morphological and structural characteristics of the 3D-AAOs and 3D-CTs with different vertical pore/tube diameters and spacing. Top-view SEM images of **a** 3D-CT-P-195 V, **b** 3D-CT-M-155 V, **c** 3D-CT-M-105 V, **d** 3D-CT-M-65 V, and **e** 3D-CT-O-50 V. The vertical **f** *D*_P_ and **g** *D*_int_ distribution diagram. **h** Plots of vertical *D*_P_ and*D*_int_ of the 3D-AAO vs. anodization voltage. **i** Typical cross-sectional SEM image of 3D-CT-M-65 V. The inset shows its transmission electron microscope (TEM) image

3D-AAO templates anodized at different voltages (denoted as O-50 V-AAO, M-55 V-AAO, M-65 V-AAO, M-85 V-AAO, M-105 V-AAO, M-125 V-AAO, M-140 V-AAO, M-155 V-AAO, and P-195 V-AAO, where the capital letters O, P, and M represent oxalic acid, phosphoric acid electrolyte, and their mixture, respectively, and the Arabic numbers represent the anodizing voltages) exhibit similar pore structures, characterized by interconnected vertical and lateral pores (Figs. S3 and S4). Interestingly, the corresponding resultant 3D-CT nanoarrays become progressively denser with the anodization voltage decrease (Fig. 2a–e). Analysis of digitally processed scanning electron microscope (SEM) images yielded the distributions of vertical pore diameter *D*_P_ and interpore distance *D*_int_, presented in Figs. 2f, g and S4i, j. For P-195 V-AAO, vertical* D*_P_ and *D*_int_ were approximately 250 nm and 450 nm, respectively. Anodization in mixed acid at 155, 105, and 65 V led to progressively smaller vertical* D*_P_ (around 200, 150, and 110 nm) and *D*_int_ values (about 350, 250, and 130 nm). O-50 V-AAO displayed the smallest vertical* D*_P_ (70 nm) and *D*_int_ (110 nm). Remarkably, the vertical pore diameter *D*_P_ and interpore distance *D*_int_ exhibit an almost linear decrease with the anodizing voltage decrease across the range from 195 to 50 V (Fig. 2h). The 3D-CT nanoarrays faithfully replicate the morphology of the 3D-AAO templates well (denoted as 3D-CT-O-50 V, 3D-CT-M-55, -65, -85, -105, -125, -140, and -155 V, and 3D-CT-P-195 V, respectively, Figs. 2i and S5, S6). Furthermore, the 3D-CT nanoarrays were characterized by Raman and X-ray photoelectron spectroscopy (XPS) spectra, displaying low D/G band and O/C intensity ratios (Fig. S7) [49]. The Braunauer–Emmett–Teller (BET) surface areas of 3D-CT-P-195 V and 3D-CT-M-65 V calculated from the nitrogen adsorption–desorption isotherms were increased from 94.1 to 253.0 m^2^ g^−1^, demonstrating that reducing the vertical* D*_P_ and *D*_int_ plays a crucial role in improving the specific surface area (Fig. S8) [50]. Compared to the previously reported 3D-CT-based electrodes, the preparation process of high-quality 3D-CACT electrodes is more efficient and straightforward.

### Electrochemical Performance

**Fig. 3:**
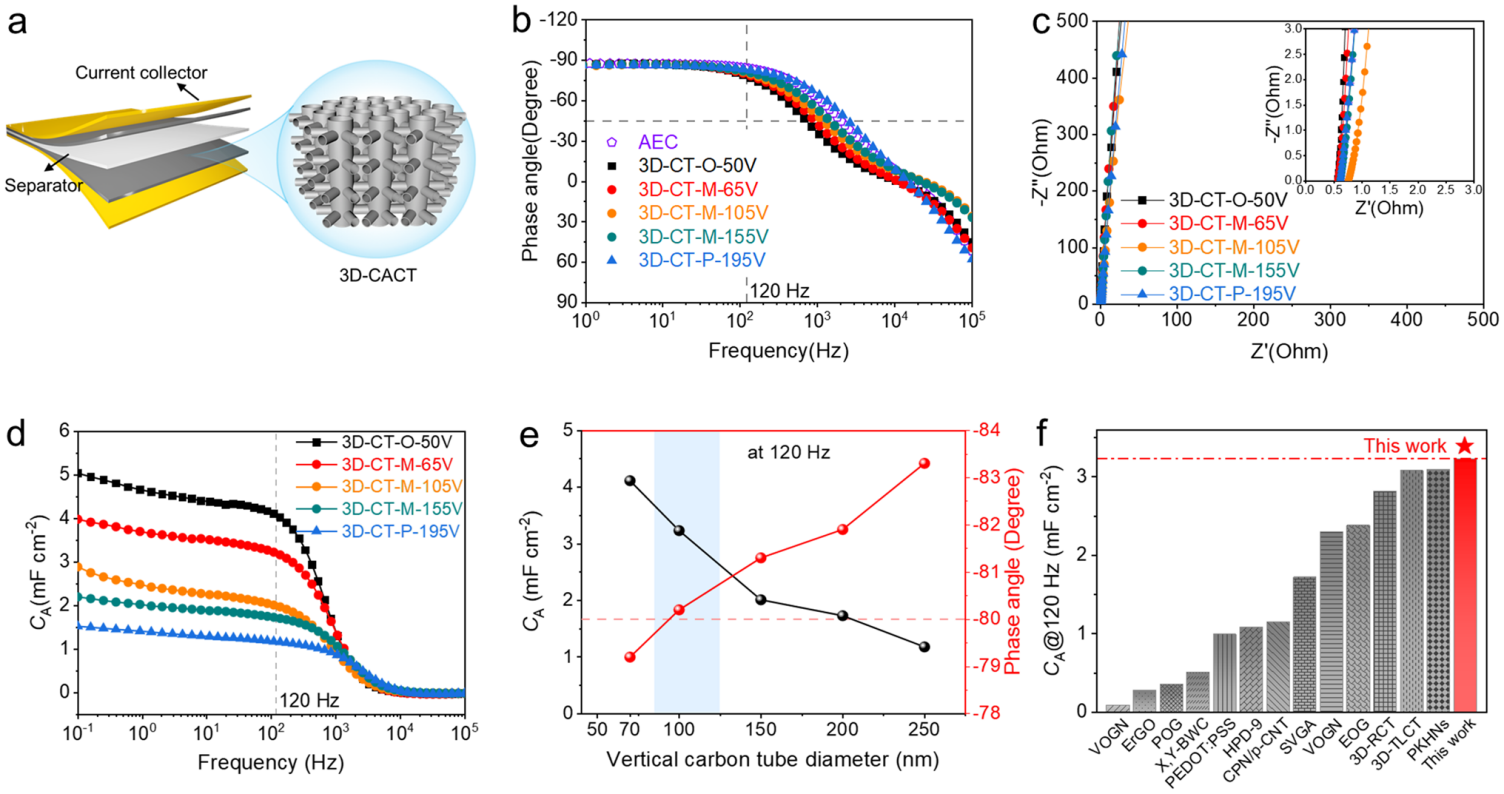
Electrochemical performances of the 3D-CT-O-50 V-, 3D-CT-M-65 V-, 3D-CT-M-105 V-, 3D-CT-M-155 V-, and 3D-CT-P-195 V-based EDLCs. **a** Schematic illustration of the 3D-CT nanoarray-based EDLC. **b** Bode plots of the above devices and AEC (330 μF/6.3 V, Panasonic, Japan). **c** Nyquist plots, the inset shows the expanded view at high frequencies. **d** Frequency-dependent areal capacitance (*C*_A_) of the 3D-CT-O-50 V-, 3D-CT-M-65 V-, 3D-CT-M-105 V-, 3D-CT-M-155 V-, and 3D-CT-P-195 V-based EDLCs. **e** Plots of *C*_A_ and phase angles at 120 Hz vs. vertical CT diameter. **f** Comparison of the* C*_A_ at 120 Hz of 3D-CT-M-65 V-based EDLC and other reported sandwich-type electrochemical capacitors used in the AC filter circuits with the phase angle near or less than − 80°

After assembling two identical 3D-CT nanarrays as electrodes and using a 1 M sulfuric acid solution as the electrolyte, along with two pieces of Pt foils as the current collectors, the symmetrical EDLCs were constructed (Fig. 3a). The 3D-CTs of 3D-CT-O-50 V, 3D-CT-M-65 V, 3D-CT-M-105 V, 3D-CT-M-155 V, and 3D-CT-P-195 V with the same thickness of approximately 12 μm were used as electrodes (Fig. S9). Finite element simulation results show that the structure of the 3D-CT can maintain good stability under the pressure of the fixture during the electrochemical performance test (Fig. S10). Electrochemical impedance spectroscopy (EIS) measurements were conducted to assess the frequency response of the 3D-CTs nanoarray-based EDLCs.

The phase angle, as a function of frequency, is plotted in Fig. 3b. The closer the phase angle reaches -90°, the more capacitive behavior the device exhibits [12, 51]. At low frequencies, all the EDLCs exhibit favorable capacitive behavior. The phase angles at 120 Hz of the 3D-CT-O-50 V-, 3D-CT-M-65 V-, 3D-CT-M-105 V-, 3D-CT-M-155 V-, and 3D-CT-P-195 V-based EDLCs achieve − 79.2°, − 80.2°, − 81.3°, − 81.9°, and − 83.3°, respectively. Most of the values of phase angles at 120 Hz are comparable to that of a commercial AEC (330 μF/6.3 V, Panasonic, Japan, Figs. 3b and S11), indicating the sufficiently fast frequency response of the 3D-CT-based EDLCs. Moreover, as the diameter of the vertical CTs decreases, the pore size in the 3D-CT nanoarray electrode also decreases, leading to a narrowing of the ion transport paths and an increase in the phase angle. The frequency at the phase angle of − 45° (*f*_-45_), recognized as the cutoff value to distinguish the capacitive and resistive nature of an EDLC [15], reaches 754, 957, 1211, 1539, and 2431 Hz for the above EDLCs (Fig. 3b), respectively. The straight lines are almost parallel to the imaginary axis at low frequencies in the Nyquist plots (Fig. 3c), revealing ideally capacitive characteristics [52]. The absence of the 45° oblique line in the low-frequency region and semicircle in the high-frequency region suggest efficient electron conduction [53, 54]. The equivalent series resistance (ESR) values were all estimated to be less than 0.07 Ω cm^2^ (the size of the electrodes is 0.3 × 0.3 cm^2^), implying excellent electric conductivity and low internal resistance of the electrodes, as well as good interfacial contact between the electrodes and current collectors [14, 17].

A series resistor–capacitor (RC) circuit model was used to simulate the device elements. The real (*C′*) and imaginary (*C′′*) capacitances of the devices, extracted from the EIS spectra, are presented in Figs. 3d and S12. The areal capacitance *C′* represents the accessible energy storage capacitance at the corresponding frequency and is used to characterize the *C*_A_. At 120 Hz, the *C*_A_ of the 3D-CT-O-50 V-, 3D-CT-M-65 V-, 3D-CT-M-105 V-, 3D-CT-M-155 V-, and 3D-CT-P-195 V-based EDLCs reaches 4.11, 3.23, 2.01, 1.73, and 1.18 mF cm^−2^, respectively. The *C*_A_ can also be obtained from the cyclic voltammetry (CV) and galvanostatic charge–discharge (GCD) tests, which show similar value magnitudes (Fig. S13). The 3D-CT-M-65 V-based EDLC exhibits a *C*_A_ value of 3.23 mF cm^−2^ at 120 Hz, almost tripling that of the 3D-CT-P-195 V-based EDLC. This dramatic increase highlights the significant impact of high-density 3D-CT nanoarray electrodes in enhancing the capacitance of line-filtering EDLCs. Notably, this *C*_A_ value is the highest among all the sandwich-type filtering EDLCs reported to date with a phase angle lower than − 80° at 120 Hz (Fig. 3e, f, Table S1). The specific volumetric capacitance *C*_V_ at 120 Hz can approach 1.71 F cm^−3^ for the 3D-CT-O-50 V electrode (Fig. S14a).

From the frequency (*f*_0_) at which the *C”* reaches the maximum value, the relaxation time constant *τ*_0_ (τ_*0*_ = 1/*f*_0_) can be derived, which represents the minimum time required for discharging with an energy efficiency of over 50% [34, 55]. The *τ*_0_ at 120 Hz is calculated to be 1.33, 1.04, 0.83, 0.65, and 0.41 ms, respectively. The relatively short time means a fast discharge characteristic. The RC time constant (*τ*_RC_) is a critical parameter reflecting the charging/discharging speed [35, 56]. The *τ*_RC_ values are measured to be 0.28, 0.25, 0.27, 0.2, and 0.17 ms at 120 Hz, comparable to AEC (0.14 ms), demonstrating the highly efficient reduction of charging/discharging time for high-rate performance and rapid frequency response. The frequency-dependent *C′/C* values are close to 1 at 120 Hz (Fig. S14b), suggesting less excessive energy loss and high actual energy storage efficiency [57]. The degree of energy loss as heat dissipation can be reflected by the evolution of the dissipation factor (*DF*) with the frequency [26]. The relatively low *DF* at 120 Hz of the EDLCs mentioned above manifests the characteristic of little energy loss (Fig. S14c).

The ideal capacitive behavior and the ultrafast ion adsorption and transport performance of the 3D-CT-M-65 V-based EDLCs were further investigated through CV and GCD tests. The near quasi-rectangular shapes of the CV curves were maintained even at the scan rate of 1000 V s^−1^, as shown in Fig. S15a, b, and a linear relationship of discharge current density with the scan rates up to 1000 V s ^− 1^ is presented (Fig. S15c), suggesting ultrafast charging/discharging and excellent rate capabilities within the electrodes. These characteristics were also verified by GCD tests (Fig. S15d–f), in which all the curves are close to triangular curves, and the derived specific capacitances show stable retention under high current densities. Furthermore, the 3D-CT-M-65 V-based EDLC exhibits excellent electrochemical cycle stability, as evidenced by the nearly 98% capacitance retention and 100% coulombic efficiency after 12,000 cycles at the current density of 10 mA cm^−2^ (Fig. S16). The phase angle at 120 Hz remains almost unchanged during the charge/discharge process.

**Fig. 4:**
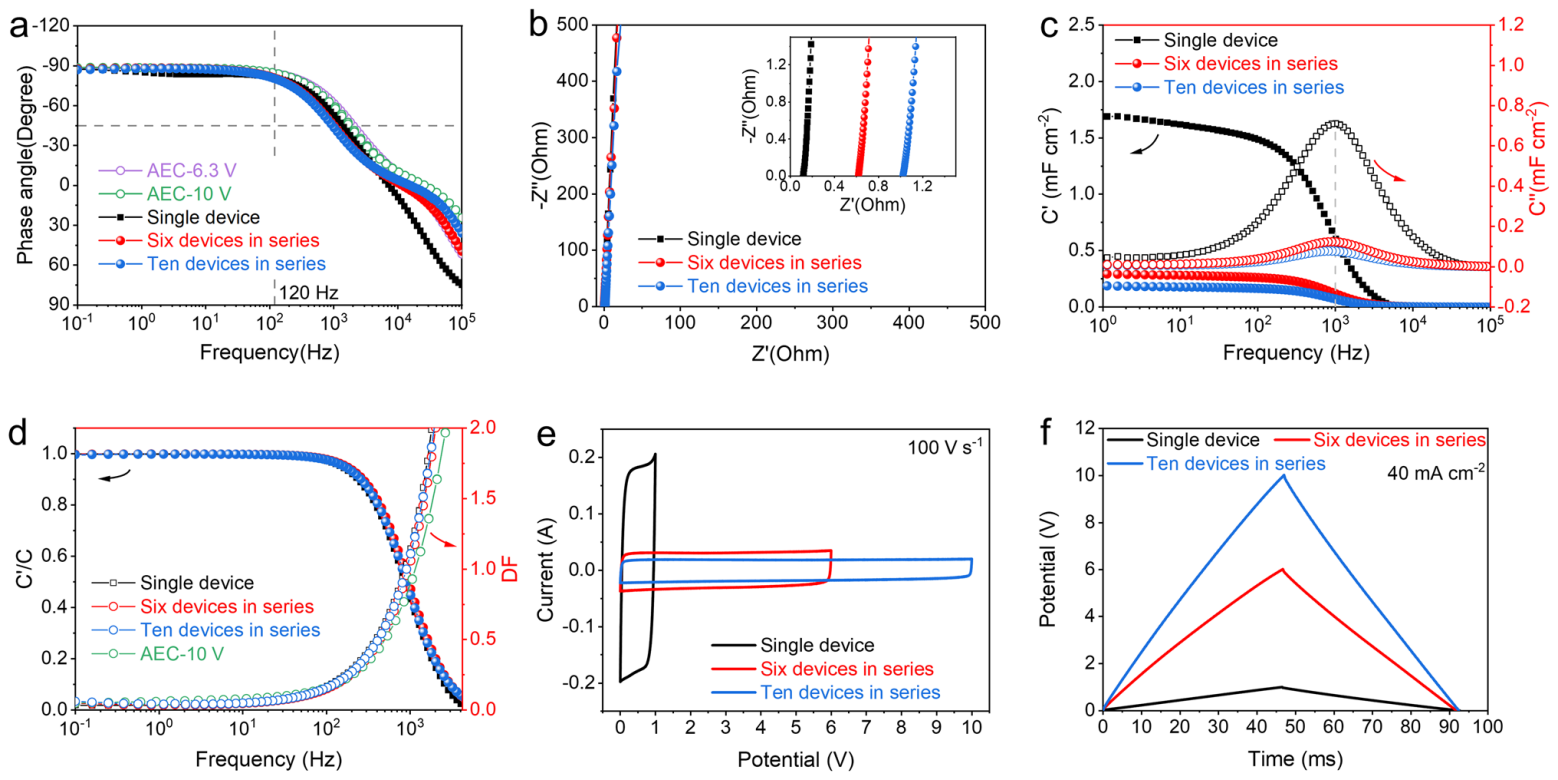
Electrochemical performances of single EDLC, six and ten EDLCs in series. **a** Bode plots. **b** Nyquist plots. **c** Real and imaginary parts of capacitance versus frequency. **d** Variations of *C′/C* and *DF* versus frequency. **e** CV curves and **f** GCD curves

To fabricate capacitor banks with high operating voltage, integrating EDLC units is critical for multifunctional practical applications [29]. Six and ten 3D-CT-M-65 V-based EDLC units, each with an electrode area of approximately 1 cm^2^ and a thickness of 8.5 μm, were connected in series to benchmark AECs operating at 6.3- and 10-V (Figs. S17 and S18). The capacitor banks exhibited stable and excellent electrochemical performance, as verified by EIS, CV, and GCD tests. Compared with the single device (− 81.2°), the phase angles of six or ten devices in series at 120 Hz increase slightly, reaching about − 81.0° and − 80.6°, respectively (Fig. 4a), indicating that the integrated capacitor banks still have excellent frequency response comparable to the rated AECs. The nearly vertical line features in the low-frequency region in the Nyquist plots indicate typical capacitive behaviors of the devices in series (Fig. 4b). The ESR of six or ten devices in series is around 6 or 10 times of a single device (Table S2).

Moreover, the six and ten EDLC units in series display almost the same relaxation time *τ*_0_ as that of the single device (Fig. 4c); this is mainly due to the stability of the structure and electrochemical properties of the nanoarray electrodes, the close electrical contact between electrodes and current collectors, and between the units. The *C*_A_ of a single device is approximately 6 and 10 times that of six and ten devices in a series. The *C’/C* values are close to 1, and the *DF* is steady at a low value at 120 Hz (Fig. 4d), suggesting the almost lossless energy and fast frequency response performance. Besides, the quasi-rectangular shapes of CV curves and symmetrical triangular shapes of GCD curves are maintained well (Fig. 4e, f), implying the high reproducibility and high power and rate capability of fabricated EDLC units under different external voltages. All of these demonstrate the stability and homogeneity of the 3D-CACT-based EDLCs for constant performance after series connection.

The operating voltage of the 3D-CT-M-65 V-based EDLC could be further broadened by increasing the number of units in series or using an organic electrolyte. The specific volumetric capacitance at rated voltage (*C*_V vol_) of the 3D-CT-M-65 V-based EDLC was compared with commercial AECs [58]. The calculated *C*_V vol_ in the aqueous electrolyte is 0.24/*V*^2^, where *V* is the voltage rating (based on the volume of the device, Supporting Information). The device with organic electrolyte also shows high capacitive and filtering performance (Figs. S19 and S20), with the *C*_V vol_ increasing to 0.96/*V*^2^. The 3D-CT-M-65 V-based EDLCs could have a higher volumetric capacitance than commercial AECs up to ~ 25 V in the aqueous electrolyte and ~ 160 V in the organic electrolyte, respectively (Fig. S21), indicating that they could replace AECs for low voltage (< 160 V) AC line-filtering.

### AC Line-Filtering Performance

**Fig. 5:**
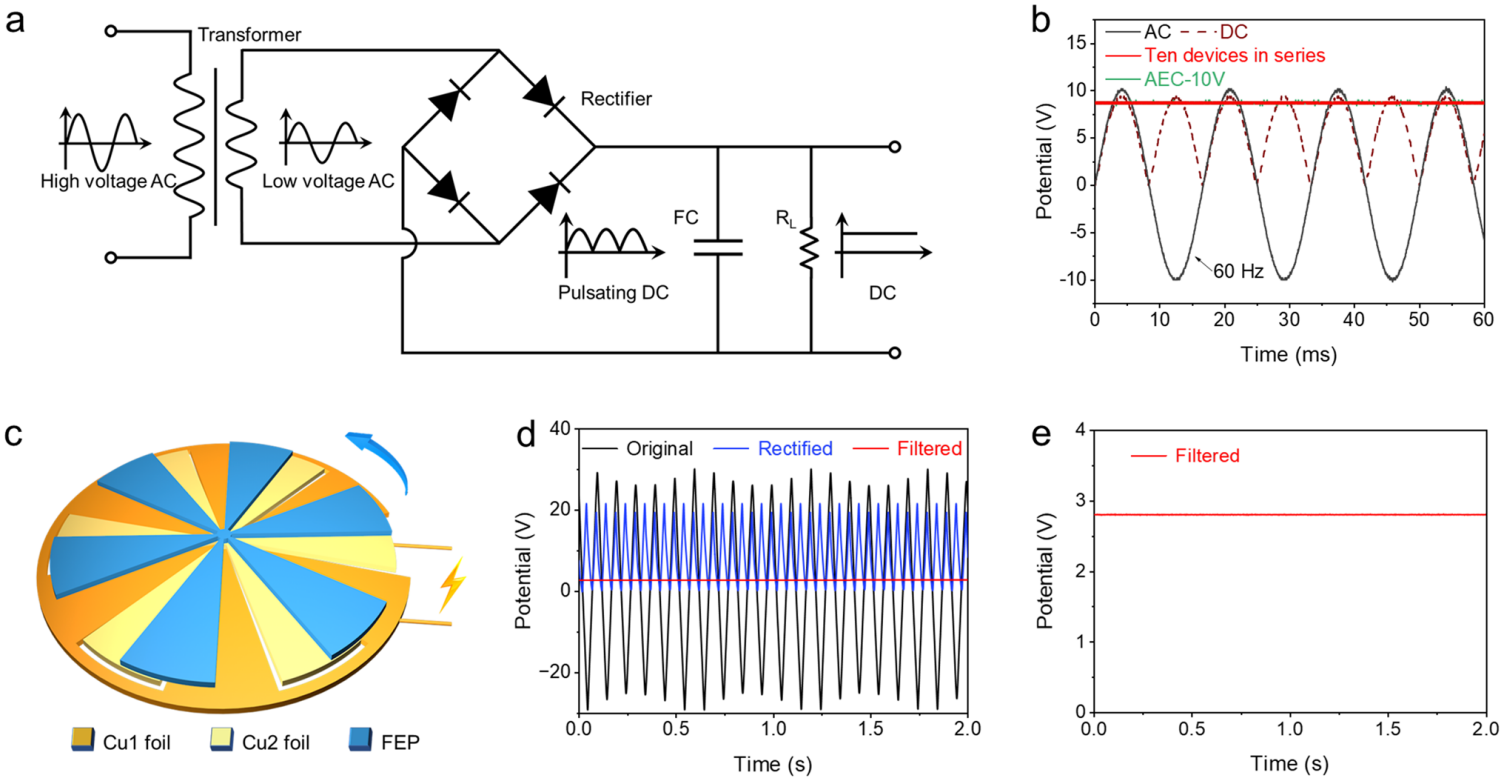
AC line-filtering performance demonstration. **a** Schematic demonstration of the rectifying and filtering circuit. The rectifier is built with four silicon Schottky diodes (1N5824, Master Instrument Corporation). **b** AC line-filtering results of the ten EDLCs in series and a commercial AEC (10 V/100 μF, Nippon, Japan) with *R*_L_ of 10 kΩ. **c** Schematic illustration of the basic structure of the RD-TENG composed of the FEP layer and the stationary Cu layer. **d** Electrical signals powered by TENG at the initial AC, rectified, and filtered DC states. **e** Filtered signal

To demonstrate realistic AC line filtering, we connected ten devices in series to filter the sinusoidal AC signal into a smooth DC signal. The filter circuit is equipped with 3D-CT-M-65 V-based EDLCs, a rectifier, and a loading resistance (*R*_L_) of 10 kΩ as illustrated in Fig. 5a. A 60 Hz AC input signal (with voltage peak to peak of 20 V) was converted to a 120 Hz DC signal via a rectifier (Fig. 5b). Subsequently, the pulsating DC signal was smoothed to a DC output with trivial voltage fluctuation after passing through the capacitors, comparable to a commercial AEC (10 V/100 μF, Nippon, Japan). The capacitors in series show comparable output voltages and small variance coefficients compared with AECs under different *R*_L_ values (Fig. S22). Furthermore, this circuit effectively filters square, triangular, arbitrary, and noise waveform ripples, demonstrating its potential for diverse filtering requirements (Fig. S23). These results indicate that the 3D-CACT-based electrode, with sufficient specific surface area, smooth ion channels, and high conductivity, can effectively facilitate ion adsorption/desorption. It shows significant potential to replace bulky AECs in AC line-filtering. In addition to AC power signals, the discontinuously pulsating AC output from a rotating disk triboelectric nanogenerator (RD-TENG) can be efficiently smoothed using the rectifying and filtering circuit with a capacitor bank. The RD-TENG consists of a stator printed with a copper pattern and a rotor made of fluorinated ethylene propylene (FEP) film (Fig. 5c) [15]. The filtering capacitor banks were achieved by connecting ten devices in series. The signals from the RD-TENG, the rectifier, and the capacitors are shown in Fig. 5d, respectively. A stable DC output voltage with almost no ripples and attenuation was obtained after rectification and filtering (Fig. 5e), demonstrating the outstanding pulse smoothing ability of the filtering capacitor banks [59]. Furthermore, the capacitors can rapidly absorb the limited charge generated by the RD-TENG, effectively safeguarding them from damage caused by transient high voltages [60]. The excellent filtering performance suggests that replacing AECs with 3D-CACT-based EDLC banks would significantly improve the practicability of miniaturizing distributed energy harvesting equipment, self-power systems, and wearable electronics.

## Conclusions

In summary, we fabricated a structurally integrated 3D-CT nanoarray with tunable vertical CT density by simultaneously regulating the vertical pore diameter *D*_P_ from 70 to 250 nm and interpore distance *D*_int_ from 110 to 450 nm of the 3D-AAO template. The resultant 3D-CACT nanoarray with smaller diameter and denser CTs exhibits significantly increased specific surface area and maintained rapid ion transport paths, leading to a high *C*_A_ of 3.23 mF cm^−2^ for sandwich-stacked EDLCs and a phase angle of -80.2° at 120 Hz, exhibiting excellent line-filtering performance. Moreover, the structure-adjustable template auxiliary method holds great potential for customizing the size of nanomaterials and developing integrated microdevices.

## Supplementary Information

Below is the link to the electronic supplementary material.

Supplementary file1 (DOCX 12651 KB)

## References

[1] P. Huang, C. Lethien, S. Pinaud, K. Brousse, R. Laloo et al., On-chip and freestanding elastic carbon films for micro-supercapacitors. Science **351**, 691–695 (2016). 10.1126/science.aad3345 26912855 10.1126/science.aad3345

[2] M.F. El-Kady, R.B. Kaner, Scalable fabrication of high-power graphene micro-supercapacitors for flexible and on-chip energy storage. Nat. Commun. **4**, 1475–1483 (2013). 10.1038/ncomms2446 23403576 10.1038/ncomms2446

[3] Y.K. Sun, Z. Chen, H.J. Noh, D.J. Lee, H.G. Jung et al., Nanostructured high-energy cathode materials for advanced lithium batteries. Nat. Mater. **11**, 942–947 (2012). 10.1038/nmat3435 23042415 10.1038/nmat3435

[4] C. Zhu, R.E. Usiskin, Y. Yu, J. Maier, The nanoscale circuitry of battery electrodes. Science **358**, eaao2808–eaao2815 (2017). 10.1126/science.aao2808 29242319 10.1126/science.aao2808

[5] W. Cheng, J. Fu, H. Hu, D. Ho, Interlayer structure engineering of mxene-based capacitor-type electrode for hybrid micro-supercapacitor toward battery-level energy density. Adv. Sci. **8**, e2100775–e2100787 (2021). 10.1002/advs.202100775 10.1002/advs.202100775 PMC8373094 34137521

[6] Y. Wang, N. Chen, B. Zhou, X. Zhou, B. Pu et al., NH_3_-induced in situ etching strategy derived 3D-interconnected porous mxene/carbon dots films for high performance flexible supercapacitors. Nano-Micro Lett. **15**, 231–242 (2023). 10.1007/s40820-023-01204-4 10.1007/s40820-023-01204-4 PMC10584800 37851182

[7] J.R. Miller, R.A. Outlaw, B.C. Holloway, Graphene double-layer capacitor with AC line-filtering performance. Science **329**, 1637–1639 (2010). 10.1126/science.1194372 20929845 10.1126/science.1194372

[8] F. Han, O. Qian, G. Meng, D. Lin, G. Chen et al., Structurally integrated 3D carbon tube grid-based high-performance filter capacitor. Science **377**, 1004–1007 (2022). 10.1126/science.abh4380 36007027 10.1126/science.abh4380

[9] Y. Hu, M. Wu, F. Chi, G. Lai, P. Li et al., Ultralow-resistance electrochemical capacitor for integrable line filtering. Nature **624**, 74–79 (2023). 10.1038/s41586-023-06712-2 37968404 10.1038/s41586-023-06712-2

[10] M. Zhao, Y. Qin, X. Wang, L. Wang, Q. Jin et al., PEDOT:PSS/Ketjenblack holey nanosheets with ultrahigh areal capacitance for kHz AC line-filtering micro-supercapacitors. Adv. Funct. Mater. (2023). 10.1002/adfm.202313495

[11] Z. Zhang, Z. Wang, F. Wang, T. Qin, H. Zhu et al., A laser-processed carbon-titanium carbide heterostructure electrode for high-frequency micro-supercapacitors. Small **19**, 2300747–2300755 (2023). 10.1002/smll.202300747 10.1002/smll.202300747 36823399

[12] F. Wang, Z. Guo, Z. Wang, H. Zhu, G. Zhao et al., Laser-induced transient self-organization of TiNx nano-filament percolated networks for high performance surface-mountable filter capacitors. Adv. Mater. **35**, 2210038–2210050 (2023). 10.1002/adma.202210038 10.1002/adma.202210038 36688671

[13] M. Zhang, K. Dong, S.S. Garakani, A.K. Kheirabad, I. Manke et al., Bridged carbon fabric membrane with boosted performance in AC line-filtering capacitors. Adv. Sci. **9**, e2105072–e2105079 (2022). 10.1002/advs.202105072 10.1002/advs.202105072 PMC8895147 35060354

[14] Y. Wen, H. Chen, M. Wu, C. Li, Vertically oriented mxene bridging the frequency response and capacity density gap for AC-filtering pseudocapacitors. Adv. Funct. Mater. **32**, 2111613–2111622 (2022). 10.1002/adfm.202111613

[15] Z. Li, X. Wang, L. Zhao, F. Chi, C. Gao et al., Aqueous hybrid electrochemical capacitors with ultra-high energy density approaching for thousand-volts alternating current line filtering. Nat. Commun. **13**, 6359–6369 (2022). 10.1038/s41467-022-34082-2 36289214 10.1038/s41467-022-34082-2 PMC9606111

[16] J. Zhang, K. Wang, P. Lu, J. Gao, Z. Cao et al., Wood-like low-tortuosity thick electrode for micro-redoxcapacitor with ultrahigh areal energy density and steady power output. Adv. Funct. Mater. **34**, 2310775–2310785 (2023). 10.1002/adfm.202310775

[17] C. Zhang, H. Du, K. Ma, Z. Yuan, Ultrahigh-rate supercapacitor based on carbon nano-onion/graphene hybrid structure toward compact alternating current filter. Adv. Energy Mater. **10**, 2002132–2002148 (2020). 10.1002/aenm.202002132

[18] M. Zhang, W. Wang, L. Tan, M. Eriksson, M. Wu et al., From wood to thin porous carbon membrane: Ancient materials for modern ultrafast electrochemical capacitors in alternating current line filtering. Energy Stor. Mater. **35**, 327–333 (2021). 10.1016/j.ensm.2020.11.007

[19] F. Chi, Y. Hu, W. He, C. Weng, H. Cheng et al., Graphene ionogel ultra-fast filter supercapacitor with 4 V workable window and 150 °C operable temperature. Small **18**, e2200916–e2200923 (2022). 10.1002/smll.202200916 35355413 10.1002/smll.202200916

[20] S. Wu, Y. Yang, M. Sun, T. Zhang, S. Huang et al., Dilute aqueous-aprotic electrolyte towards robust Zn-ion hybrid supercapacitor with high operation voltage and long lifespan. Nano-Micro Lett. **16**, 161–172 (2024). 10.1007/s40820-024-01372-x 10.1007/s40820-024-01372-x PMC10963695 38526682

[21] J. Lin, Z. Peng, Y. Liu, F. Ruiz-Zepeda, R. Ye et al., Laser-induced porous graphene films from commercial polymers. Nat. Commun. **5**, 5714–5721 (2014). 10.1038/ncomms6714 25493446 10.1038/ncomms6714 PMC4264682

[22] M. Cai, R.A. Quinlan, R.A. Quinlan, D. Premathilake, S.M. Butler et al., Fast response, vertically oriented graphene nanosheet electric double layer capacitors synthesized from C_2_H_2_. ACS Nano **8**, 5873–5882 (2014). 10.1021/nn5009319 24797018 10.1021/nn5009319

[23] Z.S. Wu, Z. Liu, K. Parvez, X. Feng, K. Mullen, Ultrathin printable graphene supercapacitors with AC line-filtering performance. Adv. Mater. **27**, 3669–3675 (2015). 10.1002/adma.201501208 25973974 10.1002/adma.201501208

[24] F. Chi, C. Li, Q. Zhou, M. Zhang, J. Chen et al., Graphene-based organic electrochemical capacitors for AC line filtering. Adv. Energy Mater. **7**, 1700591–1700597 (2017). 10.1002/aenm.201700591

[25] J. Ye, H. Tan, S. Wu, K. Ni, F. Pan et al., Direct laser writing of graphene made from chemical vapor deposition for flexible, integratable micro-supercapacitors with ultrahigh power output. Adv. Mater. **30**, e1801384–e1801391 (2018). 10.1002/adma.201801384 29774618 10.1002/adma.201801384

[26] S. Xu, Y. Wen, Z. Chen, N. Ji, Z. Zou et al., Vertical graphene arrays as electrodes for ultra-high energy density AC line-filtering capacitors. Angew. Chem. Int. Ed. **60**, 24505–24509 (2021). 10.1002/anie.202111468 10.1002/anie.202111468 34533871

[27] D. Pech, M. Brunet, H. Durou, P. Huang, V. Mochalin et al., Ultrahigh-power micrometre-sized supercapacitors based on onion-like carbon. Nat. Nanotechnol. **5**, 651–654 (2010). 10.1038/nnano.2010.162 20711179 10.1038/nnano.2010.162

[28] C. Wang, S.V. Tang, S. Qu, Z. He, B. Peng et al., Design of efficient, reliable, and wide-band filter electrochemical capacitors via matching positive with negative electrodes. Joule (2024). 10.1016/j.joule.2024.01.014

[29] M. Wu, F. Chi, H. Geng, H. Ma, M. Zhang et al., Arbitrary waveform AC line filtering applicable to hundreds of volts based on aqueous electrochemical capacitors. Nat. Commun. **10**, 2855–2863 (2019). 10.1038/s41467-019-10886-7 31253802 10.1038/s41467-019-10886-7 PMC6598994

[30] J. Lin, C. Zhang, Z. Yan, Y. Zhu, Z. Peng et al., 3-dimensional graphene carbon nanotube carpet-based microsupercapacitors with high electrochemical performance. Nano Lett. **13**, 72–78 (2013). 10.1021/nl3034976 23237453 10.1021/nl3034976

[31] Q. Li, S. Sun, A.D. Smith, P. Lundgren, Y. Fu et al., Compact and low loss electrochemical capacitors using a graphite/carbon nanotube hybrid material for miniaturized systems. J. Power. Sources **412**, 374–383 (2019). 10.1016/j.jpowsour.2018.11.052

[32] Z. Cao, H. Hu, D. Ho, Micro-redoxcapacitor: A hybrid architecture out of the notorious energy-power density dilemma. Adv. Funct. Mater. **32**, 2111805–2111814 (2022). 10.1002/adfm.202111805

[33] Z. Cao, G. Liang, D. Ho, C. Zhi, H. Hu, Interlayer injection of low-valence Zn atoms to activate MXene-based micro-redox capacitors with battery-type voltage plateaus. Adv. Funct. Mater. **33**, 2303060–2303070 (2023). 10.1002/adfm.202303060

[34] J. Park, W. Kim, History and perspectives on ultrafast supercapacitors for AC line filtering. Adv. Energy Mater. **11**, 2003306–2003333 (2021). 10.1002/aenm.202003306

[35] H. Tang, Y. Tian, Z.S. Wu, Y.J. Zeng, Y. Wang et al., AC line filter electrochemical capacitors: materials, morphology and configuration. Energy Environ. Mater. **5**, 1060–1083 (2022). 10.1002/eem2.12285

[36] S. Xu, M. Wu, J. Zhang, Ultrafast electrochemical capacitors with carbon related materials as electrodes for AC line filtering. Chemistry **28**, e202200237 (2022). 10.1002/chem.202200237 35297538 10.1002/chem.202200237

[37] H. Sun, L. Mei, J. Liang, Z. Zhao, C. Lee et al., Three-dimensional holey-graphene/niobia composite architectures for ultrahigh-rate energy storage. Science **356**, 599–604 (2017). 10.1126/science.aam5852 28495745 10.1126/science.aam5852

[38] S. Li, D. Liu, G. Wang, P. Ma, X. Wang et al., Vertical 3D nanostructures boost efficient hydrogen production coupled with glycerol oxidation under alkaline conditions. Nano-Micro Lett. **15**, 189–201 (2023). 10.1007/s40820-023-01150-1 10.1007/s40820-023-01150-1 PMC10387032 37515627

[39] J. Qiu, Y. Duan, S. Li, H. Zhao, W. Ma et al., Insights into nano- and micro-structured scaffolds for advanced electrochemical energy storage. Nano-Micro Lett. **16**, 130 (2024). 10.1007/s40820-024-01341-4 10.1007/s40820-024-01341-4 PMC10891041 38393483

[40] F. Han, G. Meng, F. Zhou, L. Song, X. Li et al., Dielectric capacitors with three-dimensional nanoscale interdigital electrodes for energy storage. Sci. Adv. **1**, e1500605–e1500611 (2015). 10.1126/sciadv.1500605 26601294 10.1126/sciadv.1500605 PMC4646808

[41] F. Han, G. Meng, Q. Xu, X. Zhu, X. Zhao et al., Alumina-sheathed nanocables with cores consisting of various structures and materials. Angew. Chem. Int. Ed. **50**, 2036–2040 (2011). 10.1002/anie.201007151 10.1002/anie.201007151 21344546

[42] W. Lee, K. Schwirn, M. Steinhart, E. Pippel, R. Scholz et al., Structural engineering of nanoporous anodic aluminium oxide by pulse anodization of aluminium. Nat. Nanotechnol. **3**, 234–239 (2008). 10.1038/nnano.2008.54 18654508 10.1038/nnano.2008.54

[43] F. Han, G. Meng, D. Lin, G. Chen, S. Zhang et al., Ultrahigh-power electrochemical double-layer capacitors based on structurally integrated 3D carbon tube arrays. Nano Res. **16**, 12849–12854 (2023). 10.1007/s12274-023-6263-0

[44] S. Zhang, F. Han, Q. Pan, D. Lin, X. Zhu et al., 3D grid of carbon tubes with Mn_3_O_4_-NPs/CNTs filled in their inner cavity as ultrahigh-rate and stable lithium anode. Energy Environ. Mater. **6**, e12586–e12593 (2023). 10.1002/eem2.12586

[45] S. Zhang, F. Han, Q. Pan, D. Lin, G. Chen et al., Enhancing electrochemical energy storage capacity and rate performance of the anode with a 3D interconnected carbon tube-NiO-SnO_2_ composite scaffold. Sci. China Mater. **66**, 3493–3500 (2023). 10.1007/s40843-023-2526-8

[46] G. Chen, F. Han, D. Lin, S. Zhang, Q. Pan et al., Three-dimensional multi-layer carbon tube electrodes for AC line-filtering capacitors. Joule **8**, 1080–1091 (2024). 10.1016/j.joule.2024.01.026

[47] S.Z. Kure-Chu, K. Osaka, H. Yashiro, H. Segawa, K. Wada et al., Controllable fabrication of networked three-dimensional nanoporous anodic alumina films on low-purity Al materials. J. Electrochem. Soc. **162**, C24–C34 (2014). 10.1149/2.0511501jes

[48] J. Vanpaemel, A.M. Abd-Elnaiem, S. De Gendt, P.M. Vereecken, The formation mechanism of 3D porous anodized aluminum oxide templates from an aluminum film with copper impurities. J. Phys. Chem. C **119**, 2105–2112 (2015). 10.1021/jp508142m

[49] Y. Fan, Z. Yi, G. Song, Z. Wang, C. Chen et al., Self-standing graphitized hybrid nanocarbon electrodes towards high-frequency supercapacitors. Carbon **185**, 630–640 (2021). 10.1016/j.carbon.2021.09.059

[50] W. Zhao, J. Yang, Y. Shang, B. Yang, D. Han et al., 3D carbon nanotube-mesoporous carbon sponge with short pore channels for high-power lithium-ion capacitor cathodes. Carbon **203**, 479–489 (2023). 10.1016/j.carbon.2022.12.009

[51] S. Suh, K. Kim, J. Park, W. Kim, Ultrafast flexible PEDOT:PSS supercapacitor with outstanding volumetric capacitance for AC line filtering. Chem. Eng. J. **463**, 142377–142386 (2023). 10.1016/j.cej.2023.142377

[52] M. Wu, K. Sun, J. He, Q. Huang, W. Zhan et al., Hierarchically 3D fibrous electrode for high-performance flexible AC-line filtering in fluctuating energy harvesters. Adv. Funct. Mater. **33**, 2305039–2305049 (2023). 10.1002/adfm.202305039

[53] Z. Li, L. Zhao, X. Zheng, P. Lin, X. Li et al., Continuous PEDOT:PSS nanomesh film: towards aqueous AC line filtering capacitor with ultrahigh energy density. Chem. Eng. J. **430**, 133012–133019 (2022). 10.1016/j.cej.2021.133012

[54] C. Li, X. Li, G. Liu, W. Yu, Z. Yang et al., Microcrack arrays in dense graphene films for fast-ion-diffusion supercapacitors. Small **19**, e2301533 (2023). 10.1002/smll.202301533 36970781 10.1002/smll.202301533

[55] X. Feng, X. Shi, J. Ning, D. Wang, J. Zhang et al., Recent advances in micro-supercapacitors for AC line-filtering performance: from fundamental models to emerging applications. eScience **1**, 124–140 (2021). 10.1016/j.esci.2021.11.005

[56] D. Zhao, K. Jiang, J. Li, X. Zhu, C. Ke et al., Supercapacitors with alternating current line-filtering performance. BMC Mater. **2**, 3–22 (2020). 10.1186/s42833-020-0009-z

[57] Z. Fan, N. Islam, S.B. Bayne, Towards kilohertz electrochemical capacitors for filtering and pulse energy harvesting. Nano Energy **39**, 306–320 (2017). 10.1016/j.nanoen.2017.06.048

[58] J.R. Miller, R.A. Outlaw, Vertically-oriented graphene electric double layer capacitor designs. J. Electrochem. Soc. **162**, A5077–A5082 (2015). 10.1149/2.0121505jes

[59] L. Wang, L. Zhao, M. Song, L. Xie, X. Wang et al., Alternatingly stacked thin film electrodes-based compact aqueous hybrid electrochemical capacitors for hundred-volts AC line filtering. J. Energy Chem. **78**, 158–168 (2022). 10.1016/j.jechem.2022.11.055

[60] H. Tang, K. Xia, J. Lu, J. Fu, Z. Zhu et al., NiTe_2_-based electrochemical capacitors with high-capacitance AC line filtering for regulating TENGs to steadily drive LEDs. Nano Energy **84**, 105931–105942 (2021). 10.1016/j.nanoen.2021.105931

