# Supplementary material for: High Density 3D Carbon Tube Nanoarray Electrode Boosting the Capacitance of Filter Capacitor
Source: Nanomicro Lett. 2024 Jul 3;16:235. doi: 10.1007/s40820-024-01458-6 (PMC11222361; doi:10.1007/s40820-024-01458-6)
Supplement: Supplementary file 1 — Supplementary file1 (DOCX 12651 KB) [file 40820_2024_1458_MOESM1_ESM.docx]

Supporting Information for

**High Density 3D Carbon Tube Nanoarray Electrode Boosting the Capacitance of Filter Capacitor**

Gan Chen^1, 2^, Fangming Han^1, 2,^ *, Huachun Ma^4^, Pei Li^1, 2^, Ziyan Zhou^1, 2^, Pengxiang Wang^1, 2^, Xiaoyan Li^4,^ *, Guowen Meng^1, 2,^ *, and Bingqing Wei^3,^ *

^1^ Key Laboratory of Materials Physics, and Anhui Key Laboratory of Nanomaterials and Nanotechnology, Institute of Solid State Physics, HFIPS, Chinese Academy of Sciences, Hefei, 230031, P. R. China

^2^ Department of Materials Science and Engineering, University of Science and Technology of China, Hefei, 230026, P. R. China

^3^ Department of Mechanical Engineering, University of Delaware, Newark, DE 19716, USA

^4^ Mechano-X Institute, Applied Mechanics Laboratory, Department of Engineering Mechanics, Tsinghua University, Beijing,100084, P. R. China

*Corresponding authors. E-mail: [gwmeng@issp.ac.cn](mailto:gwmeng@issp.ac.cn) (Guowen Meng); [weib@udel.edu](mailto:weib@udel.edu) (Bingqing Wei); [fmhan@issp.ac.cn](mailto:fmhan@issp.ac.cn) (Fangming Han); [xiaoyanlithu@tsinghua.edu.cn](mailto:xiaoyanlithu@tsinghua.edu.cn) (Xiaoyan Li)

**Supplementary Text**

- **Material characterization**

The morphologies and structures of the 3D-CT nanoarrays were investigated by field-emission scanning electron microscopy (SEM, FEI, Hitachi SU8000) and transmission electron microscope (TEM, JEOLJEM-2010). X-ray photoelectron spectroscopy (XPS) measurements were performed on a VG ESCALAB 250 spectrometer with monochromatic Al Kα (1486.71 eV) X-ray radiation (15 kV and 10 mA) and a hemispherical electron energy analyzer. Raman spectra were measured on a confocal laser micro-Raman spectrometer (Thermo Fisher DXR, USA) equipped with a He-Ne laser with an excitation of 532 nm.

- **Electrochemical measurements**

Two similar 3D-CT nanoarray electrodes were assembled with a sandwich configuration, using a non-woven separator, two platinum sheets as current collectors, and soaked in aqueous (1 M H_2_SO_4_) or organic electrolyte [1 M tetraethylammonium tetrafluoroborate (TEA-BF_4_, Sigma-Aldrich) in dry acetonitrile (Sigma-Aldrich)].

The cyclic voltammetry (CV) curves of the 3D-CT-based EDLCs were measured on a CHI 760E electrochemical station (CHI Instruments, Inc., Shanghai). The galvanostatic charge-discharge (GCD) and electrochemical impedance spectroscopy (EIS) in the frequency range of 100 mHz-100 kHz at an amplitude of 5 mV were evaluated using an electrochemical workstation (Zahzer, Zennium).

- **Calculations**

1. The frequency-dependent areal and volumetric specific capacitance of *C'* (*f*) ((*C*_A_, (F cm^-2^) and *C*_V_ (F cm^-3^)), *C"* (*f*) and resistor-capacitor time constant (*τ*_RC_), were calculated using equations (S1), (S2) and (S3), respectively.

$C'(f)=\frac{-Z''(f)}{2\pi Mf|Z(f)|^{2}}$ (S1)

$C''(f)=\frac{Z'(f)}{2\pi Mf|Z(f)|^{2}}$ (S2)

$\tau_{RC}=\frac{-Z'(f)}{2\pi f\cdot Z''(f)}$ (S3)

where *C'* (*f*) is the real part of the areal or volumetric specific capacitance; *M* is the area (*A*) or volume (*V*) of the electrode; *C"* (*f*) is the imaginary part of the areal or volumetric specific capacitance; *Z'* (*f*) or *Z"* (*f*) is the real or imaginary part of the impedance; |*Z*(*f*)| is the absolute value of the impedance.

1. The relaxation time constant *τ*_0_, the minimum time needed to discharge over 50% of all energy from the supercapacitor, used to evaluate the charge/discharge capability, was calculated by the following equation (S4).

$\tau_{0}=\frac{1}{f_{0}}$ (S4)

where the *f*_0_ is the frequency when the *C"* (*f*) reaches its maximum value.

1. The dissipation factor (*DF*) with the frequency used to demonstrate high-frequency response behavior was calculated using equation (S5).

$DF=\frac{|P|}{|Q|}=\frac{C''(f)}{C'(f)}$ (S5)

where the |*𝑃*| and |*Q*| are real and reactive power components, respectively.

1. The energy density *E* (mWh cm^-3^) and power density *P* (W cm^-3^) based on the devices were calculated from the following equations (S6) and (S7).

$E=\frac{C_{S}\cdot(\Delta V)^{2}}{2h}\times\frac{1}{3600}$ (S6)

$P=\frac{E}{\Delta t}\times3600$ (S7)

where the *C*_S_ is the specific areal capacitance, the Δ*V* is the operating voltage, the *h* is the thickness of two electrodes, and the Δ*t* is the discharge time.

1. The capacitive reactance (*X*_C_) was calculated by the equation (S8):

$X_{C}=\frac{1}{2\pi f\cdot C}$ (S8)

where *f* is the frequency, and *C* is the capacitance of EDLC.

1. The volumetric capacitance at rated voltage (*C*_V vol_) of the 3D-CACT-based SCs and the commercial AECs were calculated by the equation (S9):

$C_{V\text{vol}}=\frac{C_{d}}{v_{d}}$ (S9)

where *C*_V vol_ is the capacitance per volume at voltage rating, *C*_d_ is the capacitance of a device of the 3D-CACT-based SC or a commercial AEC, and *v*_d_ is the volume of the device with all components (the packages calculated ignore the parts beyond the device area).

The *C*_V vol_ for the 3D-CACT-based EDLC was evaluated based on the volume of the device, including all the components, such as electrodes (thickness of each electrode: ≈12 µm), a separator (thickness: 30 µm), current collectors (thickness of each Pt foils: 20 µm) and the packing PET films (total thickness: 40 µm).

For example, the areal capacitance of 3D-CACT-based SC with aqueous electrolyte is about 3.23 mF cm^-2^ (the thickness of 3D-CACT is 12 µm), and then the capacitance of the device is 0.00323A F, the volume of the device is about 0.0134A cm^3^ (the thickness of the device is 0.0134 cm, A is the area). To achieve a higher voltage, the SCs need to be connected in series. In this case, the number of the series devices, x is *V*_r_ (*V*_r_/*V*_EDLC_, *V*_r_ is the rating voltage, and *V*_EDLC_ is 1 volt). Therefore, *C*_V vol_ is about 0.24/*V*^2^ (F cm^-3^).

For the organic electrolyte (tetraethylammonium tetrafluoroborate in acetonitrile), the areal capacitance of 3D-CACT-based SC is about 2.06 mF cm^-2^ (the thickness of the 3D-CACT is 12 µm), and then the capacitance of the device is 0.00206A F, the volume of the device is about 0.0134A cm^3^ (the thickness of the device is 0.0134 cm, A is the area). For a rating voltage (*V*_r_), the number of the series devices x is *V*_r_ /2.5 (*V*_EDLC_ is 2.5 volts). Therefore, *C*_V vol_ is about 0.96/V^2^ (F cm^-3^).

- **Finite element simulations**

To investigate the stability of the 3D-CT under clamping pressure during the electrochemical performance tests, we performed a series of finite element simulations for the uniaxial compression of the CT arrays with and without lateral CTs. The simulated models of the 3D-CT were first constructed based on experimental observations and characterizations. The dimensions of the vertical and lateral CTs and the spacing between the vertical CTs were selected to correspond with the measurements obtained from the experiments closely. The vertical CTs had an outer diameter of 250 nm and an inner diameter of 230 nm. The spacing between the vertical CTs was set at 450 nm. The lateral CTs had an outer diameter of 100 nm and an inner diameter of 80 nm. The overall height of the 3D-CT was 1180 nm. For comparison, we also constructed models with no lateral CTs equipped with one layer or two layers of lateral CTs. During simulations, the models were only subjected to axial loading. The symmetric array structure of the models was considered, so the models were simplified by applying symmetric boundary conditions on the cross-sections. A linear elastic constitutive relationship and quasi-static loading conditions were utilized. The elastic modulus and Poisson's ratio of the CTs were set as 500 GPa and 0.275, respectively [S1]. Compressive loads were set along the vertical axis of the CTs for all models by controlling the displacement of the top and bottom surfaces. After simulations, the load-displacement curves were plotted.

**Supplementary Figures**


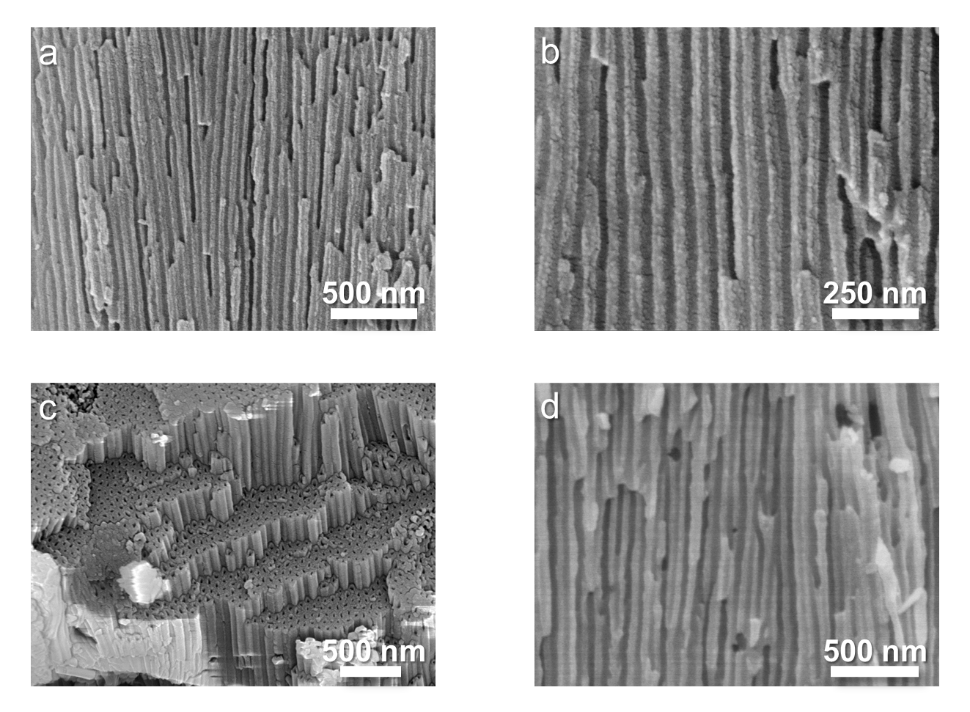


**Fig. S1** Morphological characterization of the AAO templates anodized in sulphuric acid. **a** The cross-sectional and **b** magnified SEM images of the AAO anodized in 0.3 M sulphuric acid at 25 V. **c** Top-view and **d** cross-section SEM images of the AAO anodized in 0.3 M sulphuric acid at 30 V


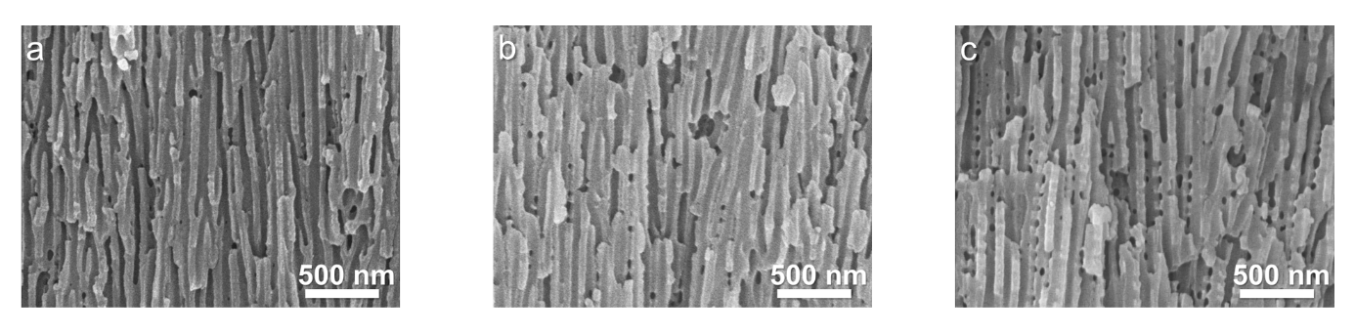


**Fig. S2** Cross-sectional SEM images of the AAO templates anodized in 0.3 M oxalic acid at **a** 40, **b** 45, and **c** 50 V


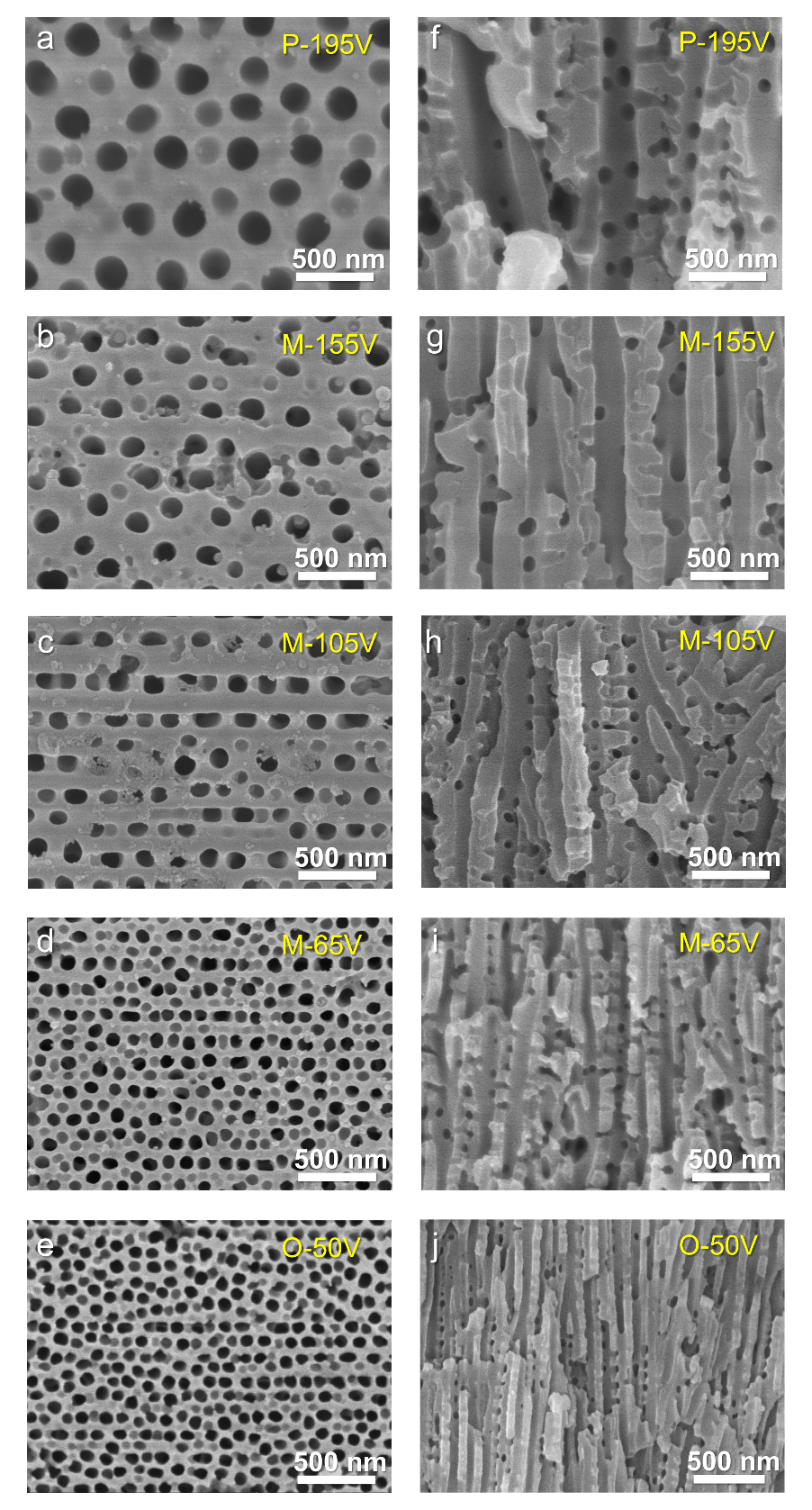


**Fig. S3** Morphological and structural characteristics of the 3D-AAO templates with different vertical *D*_P_ and *D*_int_. Top view SEM images of **a** P-195 V-AAO, **b** M-155 V-AAO, **c** M-105 V-AAO, **d** M-65 V-AAO, and **e** O-50 V-AAO. Cross-sectional SEM images of **f** P-195 V-AAO, **g** M-155 V-AAO, **h** M-105 V-AAO, **i** M-65 V-AAO, and **j** O-50 V-AAO


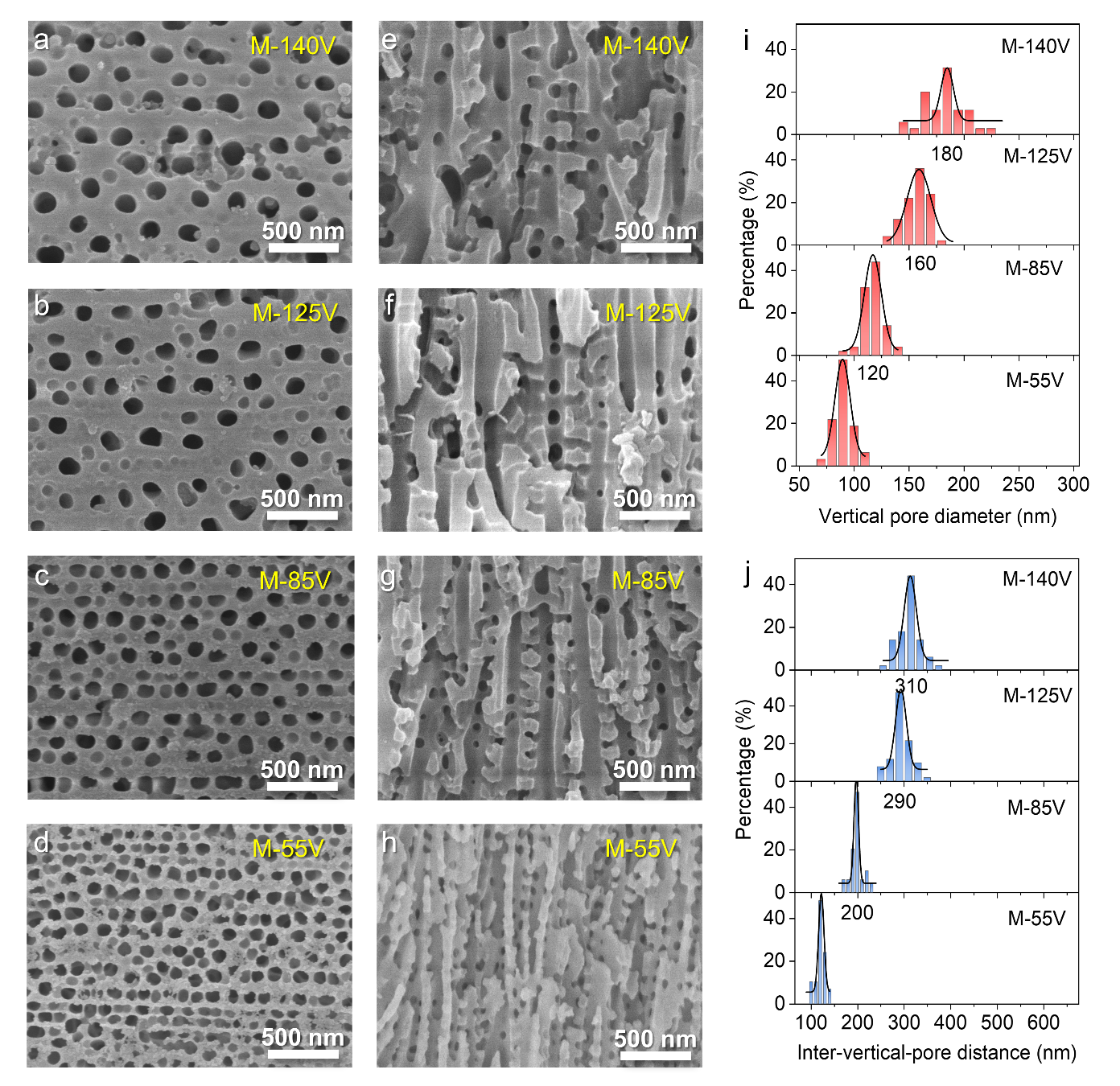


**Fig. S4** Morphological and structural characteristics of the 3D-AAO templates with different vertical *D*_P_ and *D*_int_. Top view SEM images of **a** M-140 V-AAO, **b** M-125 V-AAO, **c** M-85 V-AAO, and **d** M-55 V-AAO. Typical cross-sectional SEM images of **e** M-140 V-AAO, **f** M-125 V-AAO, **g** M-85 V-AAO, and **h** M-55 V-AAO. The vertical **i** *D*_P_ and **j** *D*_int_ distribution diagrams


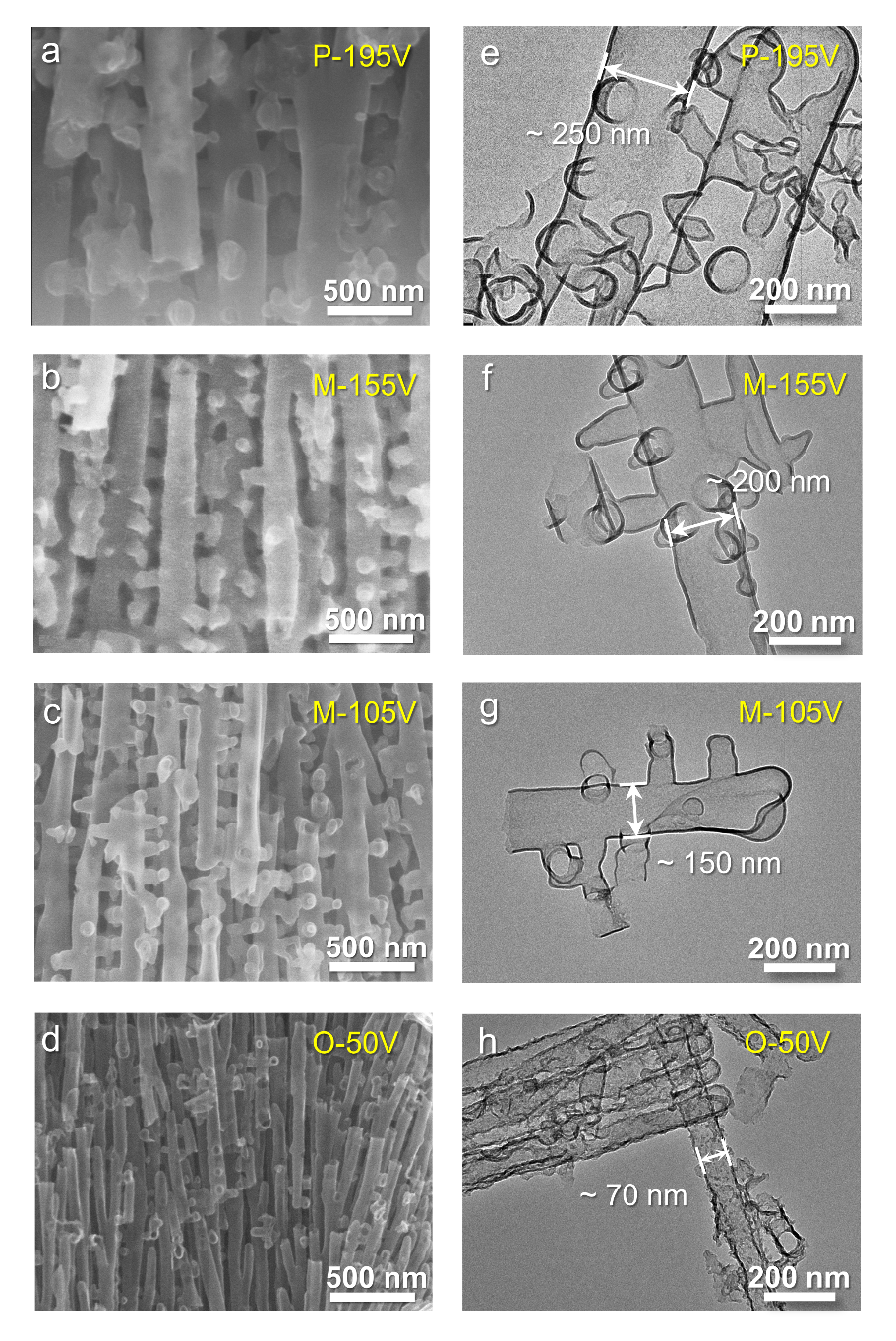


**Fig. S5** Morphological and structural characteristics of the 3D-CT nanoarrays with different vertical CT diameters and spacing. Typical cross-sectional SEM images of **a** 3D-CT-P-195 V, **b** 3D-CT-M-155 V, **c** 3D-CT-M-105 V, and **d** 3D-CT-O-50 V. TEM images of **e** 3D-CT-P-195 V, **f** 3D-CT-M-155 V, **g** 3D-CT-M-105 V, and **h** 3D-CT-O-50 V


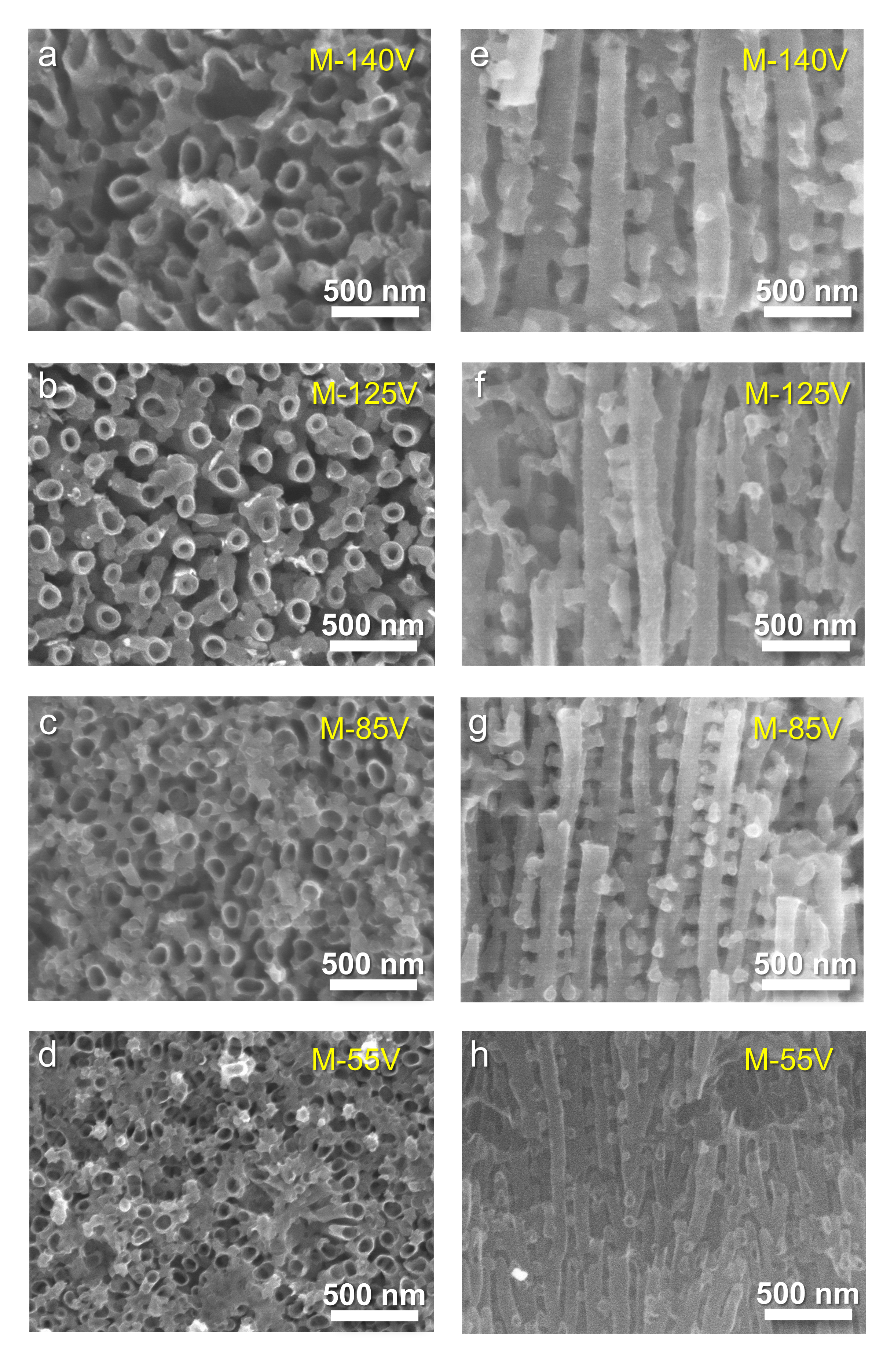


**Fig. S6** Morphological and structural characteristics of the 3D-CT nanoarrays with different vertical CT diameters and spacing. Top-view SEM images of **a** 3D-CT-M-140 V, **b** 3D-CT-M-125 V, **c** 3D-CT-M-85 V, and **d** 3D-CT-M-55 V. Cross-sectional SEM images of **e** 3D-CT-M-140 V, **f** 3D-CT-M-125 V, **g** 3D-CT-M-85 V, and **h** 3D-CT-M-55 V


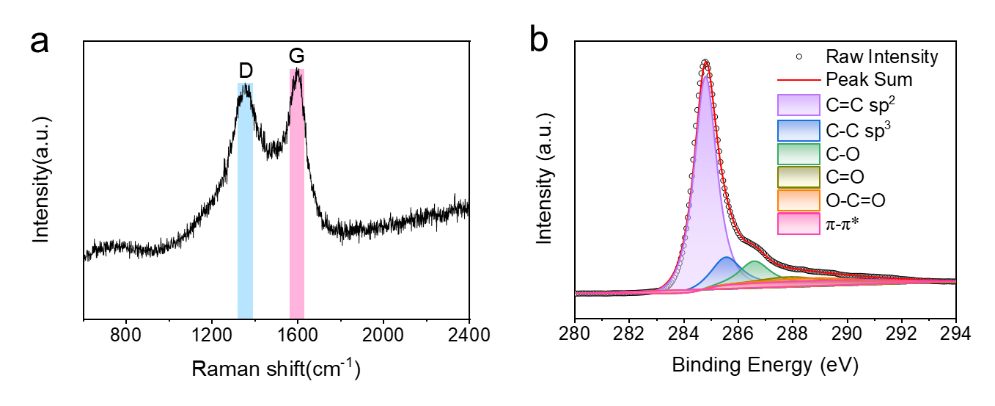


**Fig. S7** Materials characterizations of the 3D-CACT samples. **a** Raman and **b** XPS spectrum of 3D-CACT film


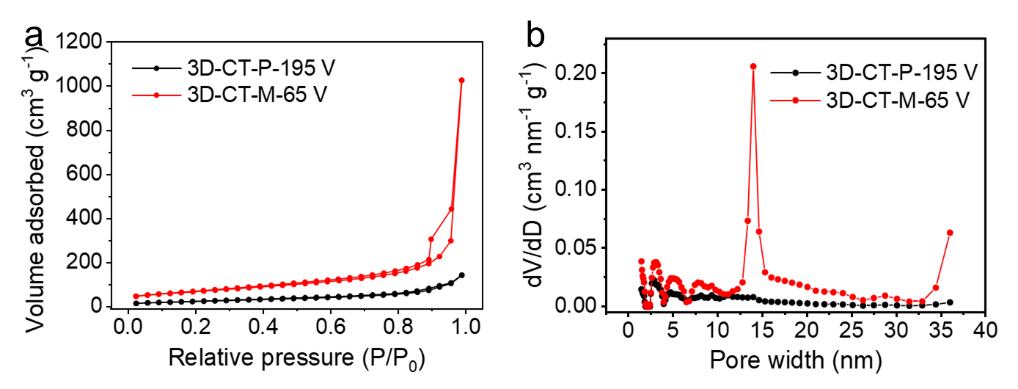


**Fig. S8** **a** Nitrogen adsorption-desorption isotherms. **b** Pore size distributions of the 3D-CT-P-195 V and 3D-CT-M-65 V


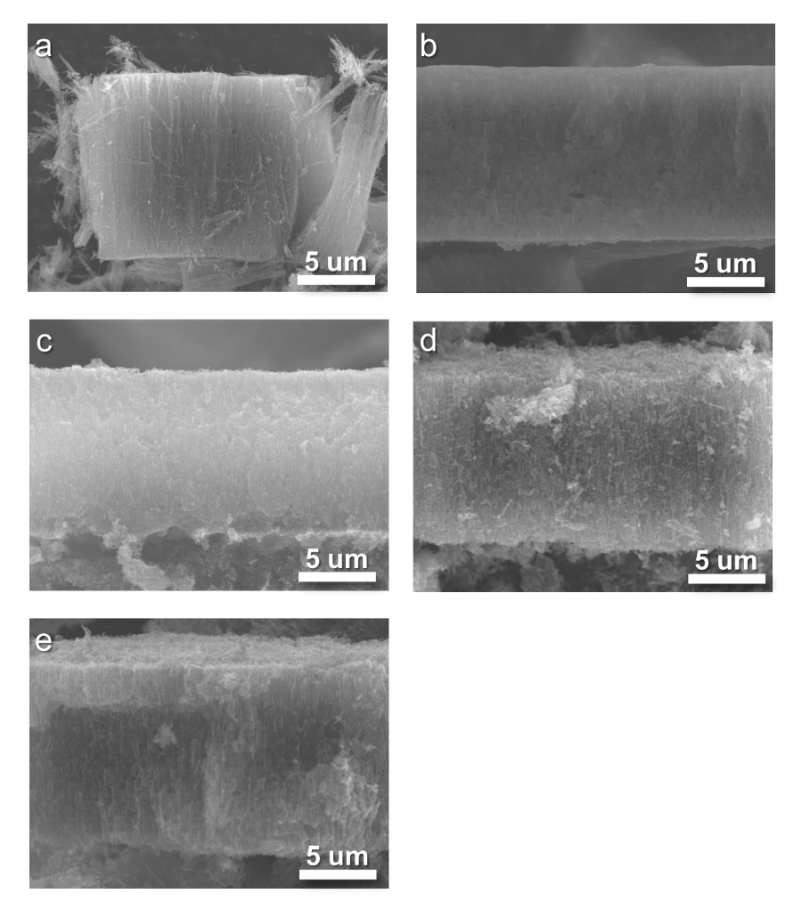


**Fig. S9** SEM images of **a** 3D-CT-O-50 V, **b** 3D-CT-M-65 V, **c** 3D-CT-M-105 V, **d** 3D-CT-M-155 V, and **e** 3D-CT-P-195 V films with a similar thickness of ~12 μm


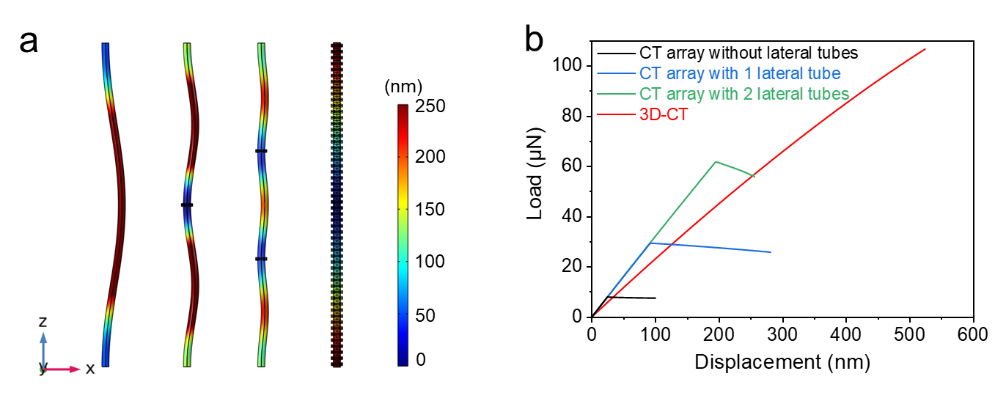


**Fig. S10** Results from finite element simulations. **a** Compressed configurations of the CT arrays without lateral CTs, CT arrays with 1 layer of lateral CTs, 2 layers of lateral CTs, and the 3D-CT. The color represents the displacement magnitude of CT. **b** Load-displacement curves of the above four models

Discussions: Finite element simulations were conducted to investigate the stability of the CT arrays with and without lateral CTs subjected to uniaxial compression. The details of simulations are supplied in the Supplementary Text. The load acting along the axial direction and the symmetry of the CT arrays ensure the simplification of the structure into a single vertical CT model (Fig. S10a). The load-displacement curves (Fig. S10b) revealed that the model without lateral CTs exhibits a critical buckling load of approximately 7.7 μN. With the addition of lateral CTs, the buckling load of the 3D-CT array increased to >100 μN. The presence of lateral CTs significantly reduced the slenderness ratio of the vertical CTs (effective slenderness ratio less than 1), preventing buckling and maintaining the structure's stability under higher loads. The slightly lower stiffness of the 3D-CT may be attributed to the holes in vertical CTs caused by the lateral CTs. These results from finite element simulations indicated the better stability of the 3D-CT structure, primarily due to the supportive effect of the lateral CTs.


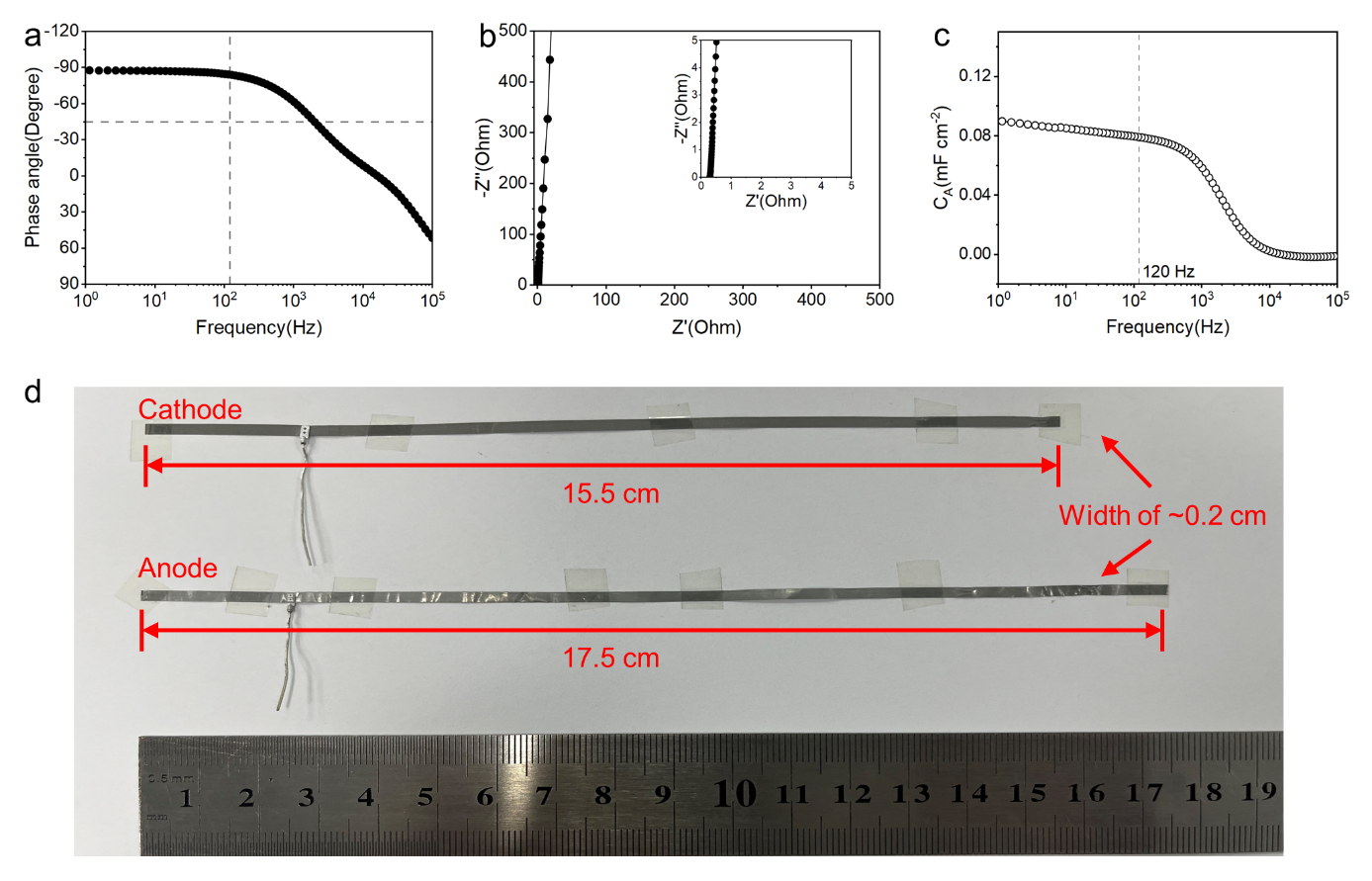


**Fig. S11** Electrochemical performances and disassembled photo of an AEC (rated capacitance of 330 μF with a working voltage of 6.3 V, Panasonic Japan). **a** Bode phase diagram, **b** Nyquist plot, and **c** Frequency-dependent *C*_A_. **d** Photograph of the cathode and anode

Discussions: Figure S11a, b shows that the AEC has a phase angle of ~-84.3° at 120 Hz and an ESR of 0.27 Ω. Figure S11d shows that the electrode of the AEC has a width of ~0.2 cm and a length of ~17.5 cm. Its *C*_A_ is calculated to be 0.08 mF cm^-2^ (Fig. S11c).


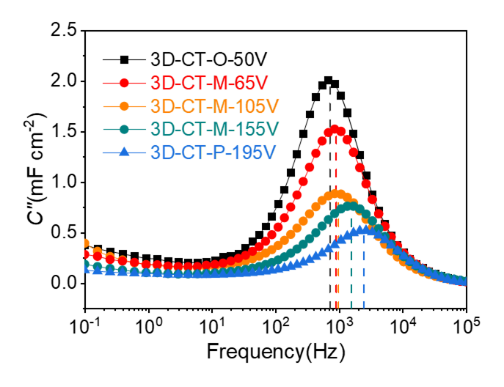


**Fig. S12** Plots of the imaginary part of the specific capacitance (C") versus frequency based on the series-RC circuit model


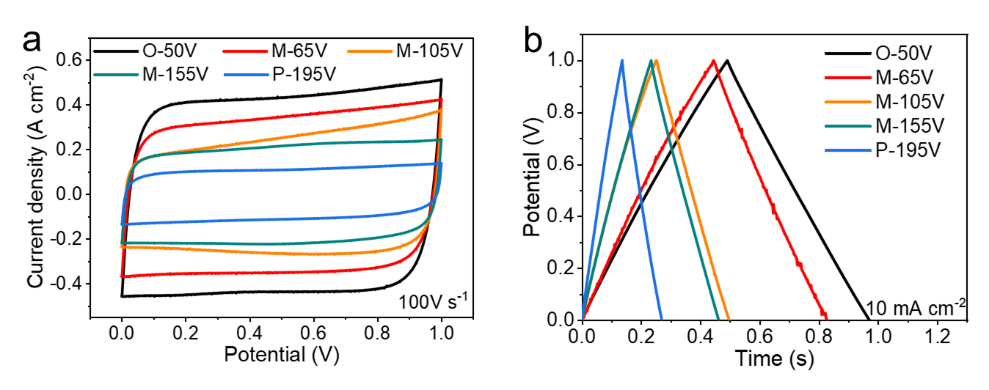


**Fig. S13** Electrochemical performances of the 3D-CT-O-50 V-, 3D-CT-M-65 V-, 3D-CT-M-105 V-, 3D-CT-M-155 V-, and 3D-CT-P-195 V-based EDLCs. **a** CV curves measured at a scan rate of 100 V s^-1^. **b** GCD curves measured at a current density of 10 mA cm^-2^


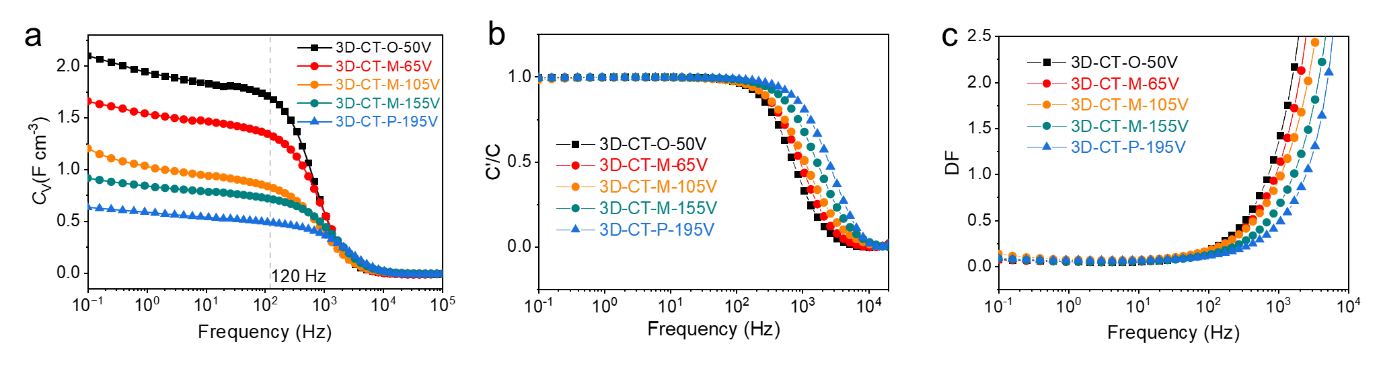


**Fig. S14** Electrochemical performances of the 3D-CT-O-50 V-, 3D-CT-M-65 V-, 3D-CT-M-105 V-, 3D-CT-M-155 V-, and 3D-CT-P-195 V-based EDLCs. **a** Frequency-dependent *C*_V_ plots. **b** The variation of C’/C and **c** dissipation factor versus frequency

Discussions: The *C*_V_ at 120 Hz of the 3D-CT-O-50 V-, 3D-CT-M-65 V-, 3D-CT-M-105 V-, 3D-CT-M-155 V-, and 3D-CT-P-195 V-based EDLCs can achieve 1.71, 1.35, 0.84, 0.72, and 0.49 F cm^-3^, respectively.


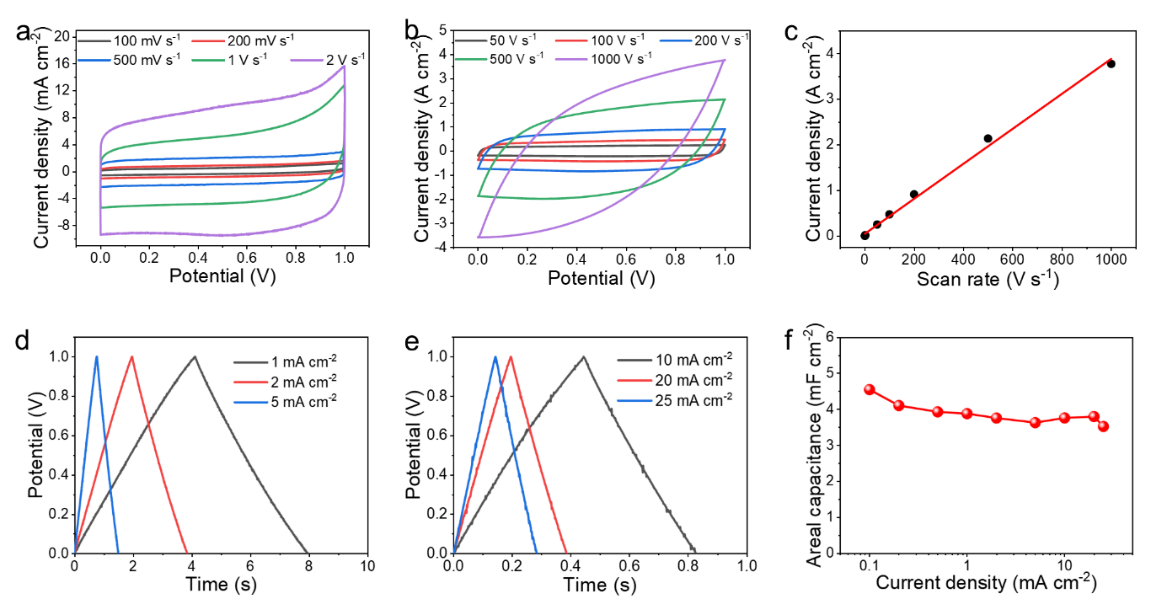


**Fig. S15** Cyclic voltammetry (CV) and galvanostatic charge/discharge (GCD) characterizations of the 3D-CT-M-65 V-based EDLC. **a, b** CV curves of the 3D-CT-M-65 V-based EDLC at the scan rates from 0.1 to 1000 V s^-1^. **c** Plot of discharge current density versus scan rate. **d, e** GCD curves measured at different current densities from 1 to 25 mA cm^-2^. **f** *C*_A_ of 3D-CT-M-65 V-based EDLC versus discharge current density


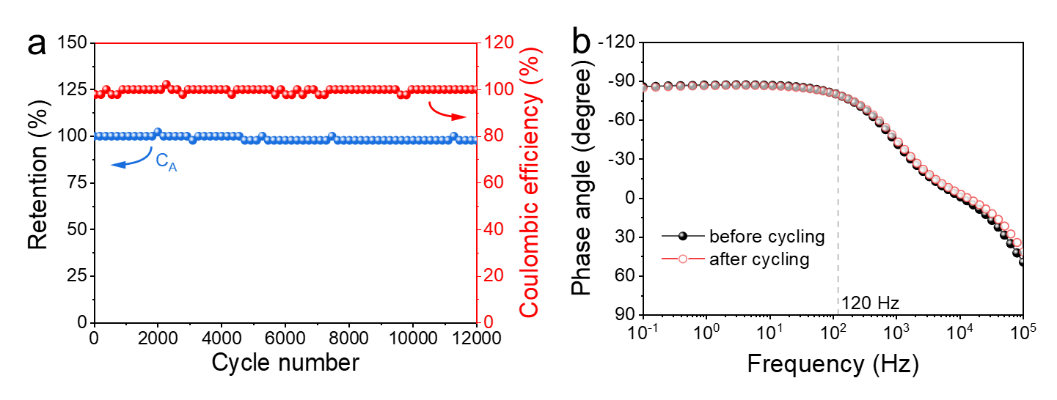


**Fig. S16** Cyclic performance characterization of the 3D-CT-M-65 V-based EDLC. **a** Electrochemical stability measurements, including retention of areal-specific capacitance and Coulombic efficiency versus charging/discharging cycles at a current density of 10 mA cm^−2^. **b** Bode plot before and after the cycling process


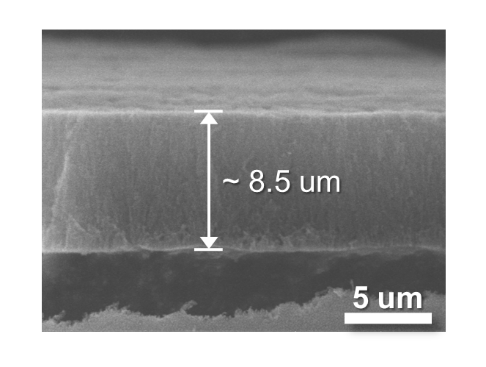


**Fig. S17** An SEM image of the 3D-CT-M-65 V film with a thickness of 8.5 μm


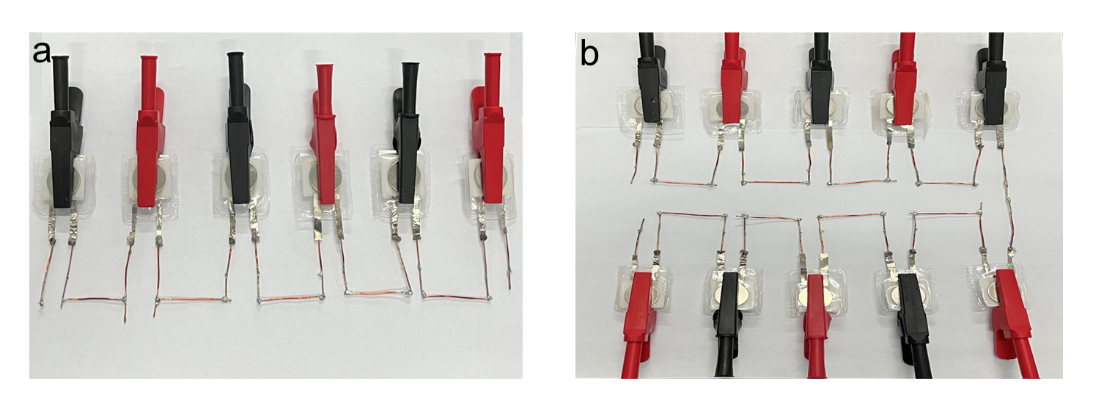


**Fig. S18** Optical images of **a** six and **b** ten EDLCs (electrode area of ~1 cm^2^) in series. The EDLCs are held in place by clamps and connected in series by copper wires


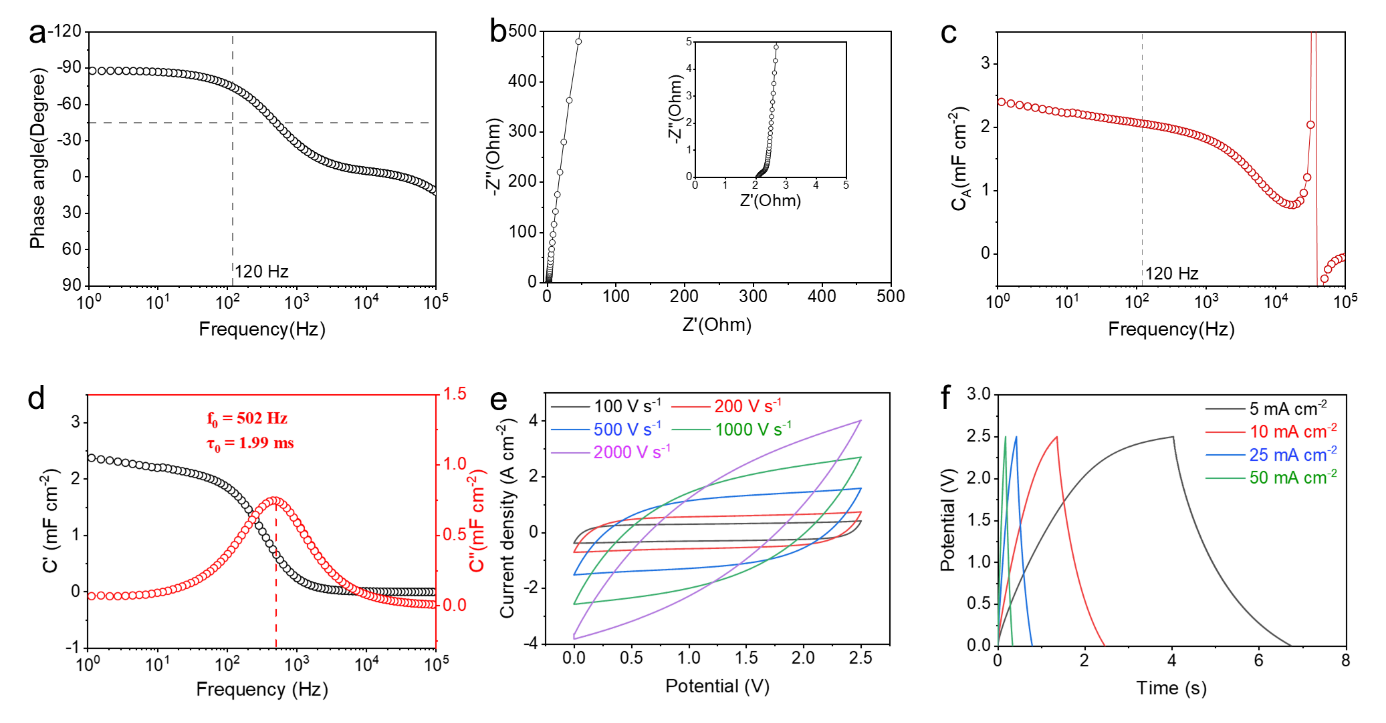


**Fig. S19** Electrochemical performances of 3D-CT-M-65 V-based EDLC in organic electrolyte. **a** Bode phase diagram. **b** Nyquist plot. **c** Specific areal capacitance. **d** Plot of C' and C" versus frequency. **e** CV curves measured at different scan rates of 100-2000 V s^-1^. **f** GCD curves measured at different current densities of 5-50 mA cm^-2^

Discussions: The electrochemical performances of the 12 μm-thick 3D-CT-M-65 V-based EDLC in an organic electrolyte were measured. At 120 Hz, the EDLC in organic electrolyte shows a phase angle of -74.7°. (Fig. S19a). The ESR and *C*_A_ are exanimated to be 2.1 Ω and 2.06 mF cm^-2^, respectively (Fig. S19b, c). The reduced capacitance and relatively slower frequency response are mainly due to the larger ionic radius in the organic electrolyte. The 3D-CT-M-65 V-based EDLC in an organic electrolyte shows a *f*_0_ of 502 Hz and a *τ_0_* of 1.99 ms (Fig. S19d). Meanwhile, power performance in organic electrolytes is also demonstrated by CV measurements with a potential window of 2.5 V. Figure S19e reveals that the capacitance characteristics of 3D-CT-M-65 V-based EDLC can be maintained well at a scan rate of 1000 V s^-1^. The IR drop is negligible over the wide potential range shown in the GCD curves (Fig. S19f), resulting from the high conductivity of the 3D-CACT and good interfacial contact between the electrodes and current collectors. Compared to measuring in aqueous electrolytes, the GCD curves show observably lower coulombic efficiency at low current densities.


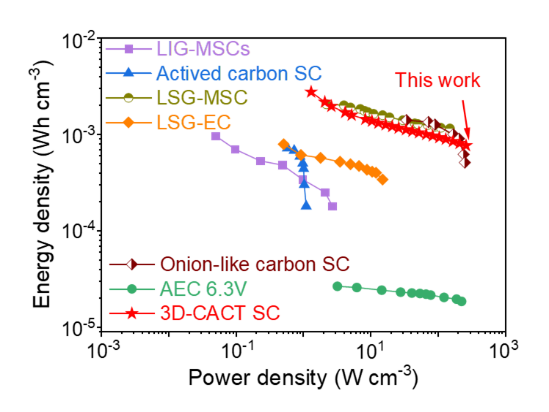


**Fig. S20** Comparison of energy and power densities of 3D-CT-M-65 V-based EDLC with those of reported AC-line filtering electrochemical capacitors in organic electrolyte

Discussions: The energy density (*E*) and power density (*P*) of the 3D-CT-M-65 V-based EDLC are assessed to evaluate its overall electrochemical performance. Notably, the Ragone plots confirm that with fast ion transport behavior in the compactly arranged carbon tube nanoarray structure, the 3D-CT-M-65 V-based EDLC with organic electrolyte has a power density of 520 W cm^-3^ while maintaining a high volumetric energy density of 8.8×10^-4^ Wh cm^-3^ (48 times that of AEC (~ 1.8×10^-5^ Wh cm^-3^)), showing its potential for compact AC line filter.


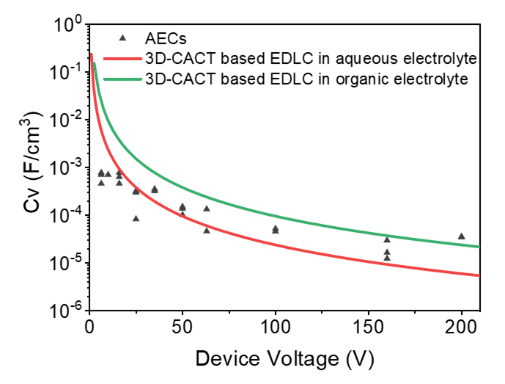


**Fig. S21** A volumetric comparison of 3D-CACT-based EDLCs with commercial AECs (red triangles; Panasonic, Nippon, Rubycon, and Nichicon, Japan).


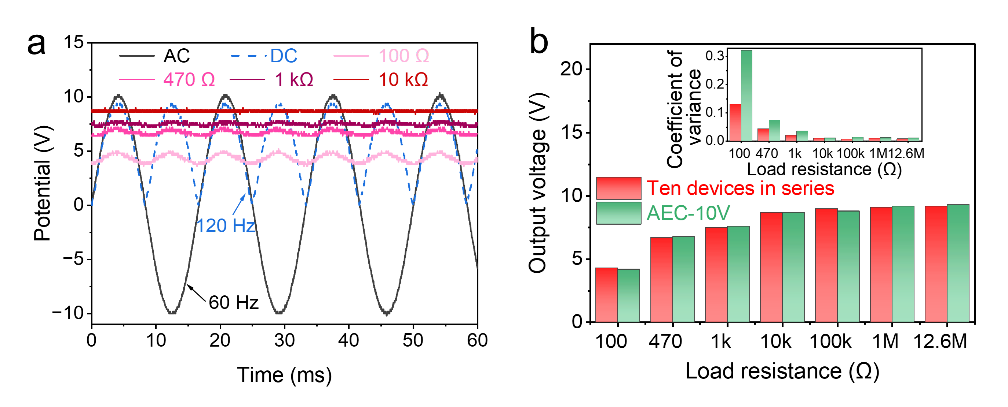


**Fig. S22 a** Output DC signals with four different loads. **b** Comparison of AC line-filtering performance of the ten EDLCs in series with AEC under different *R*_L_ values at 60 Hz. Inset compares the coefficient of variance of the ten EDLCs in series with AEC under different *R*_L_ values at 60 Hz


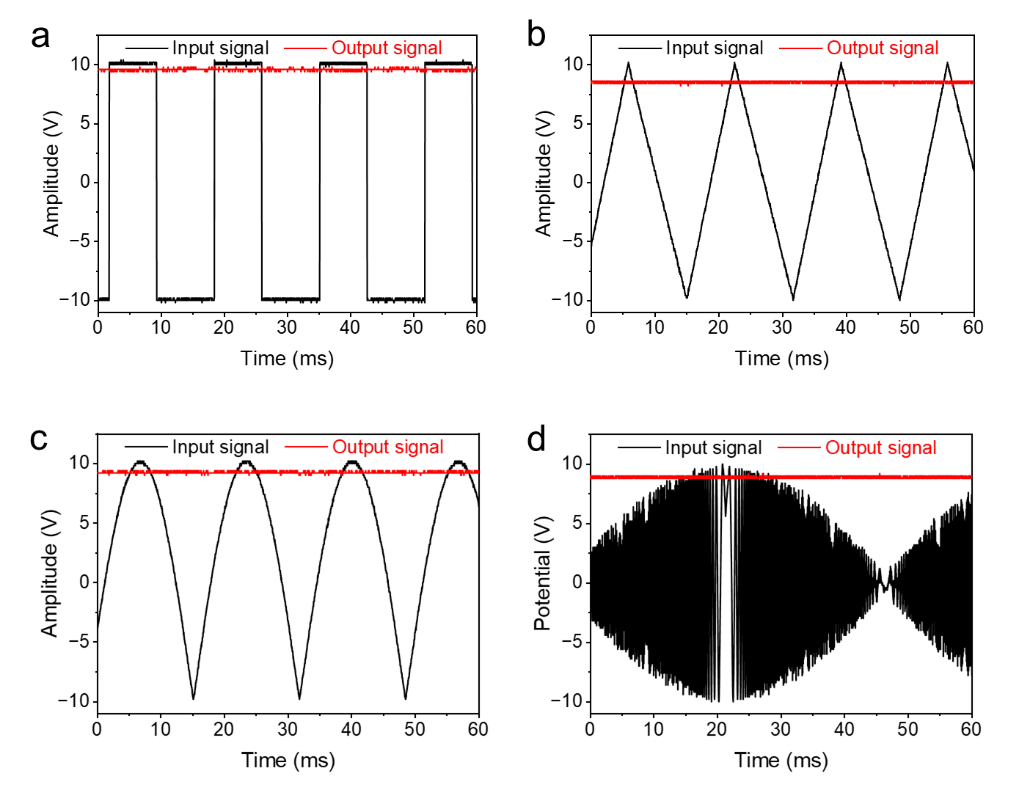


**Fig. S23** Filtering performances of the 3D-CT-M-65 V-based EDLCs for various waveforms. The AC input signals are **a** square, **b** triangular, **c** arbitrary, and **d** noise waveform

**Supplementary Tables**

**Table S1** Comparison of EDLCs based on 3D-CACT film electrodes with AEC (6.3 V/330 µF) and other reported electrochemical capacitors used in the AC filter circuits in terms of materials, capacitance, and frequency response

| Electrodes | Thickness  (μm) | Phase angle at 120 Hz (°) | *f*_-45_  (Hz) | *C*_A_ at 120 Hz (mF cm^-2^) | Type | References |
| --- | --- | --- | --- | --- | --- | --- |
| VOGN | 0.6 | -82 | 15000 | 0.09 | Sandwich | Miller *et al*. [S2] |
| ErGO | 20 | -84 | 4200 | 0.28 | Sandwich | Sheng *et al*. [S3] |
| POG | NA | -82 | 4000 | 0.36 | Sandwich | Ren *et al*. [S4] |
| X, Y-BWC | 23.3 | -83.5 | 2479 | 0.51 | Sandwich | Zhang *et al*. [S5] |
| PEDOT: PSS | NA | -83.6 | 1700 | 1.00 | Sandwich | Zhang *et al*. [S6] |
| HPD-9 | 0.25 | -84 | 1776 | 1.09 | Sandwich | Li *et al*. [S7] |
| CPN/p-CNT | 0.2 | -83.3 | 2153 | 1.15 | Sandwich | Li *et al*. [S8] |
| G/VACNTs | 20 | -84.8 | 1980 | 1.38 | Planar | Li *et al*. [S9] |
| SVGA | 6 | -80.6 | 1001 | 1.72 | Sandwich | Xu *et al*. [S10] |
| VOGN | 2.5 | -85 | NA | 2.3 | Sandwich | Premathilake *et al*. [S11] |
| EOG | 10.3 | -80.6 | 1010 | 2.34 | Sandwich | Li *et al*. [S12] |
| 3D-RCT | 12 | -80.5 | 1120 | 2.81 | Sandwich | Han *et al*. [S13] |
| 3D-TLCT | 12 | -80.1 | 951 | 3.08 | Sandwich | Chen *et al*. [S14] |
| PKHNs | 0.6 | -81.9 | 1210 | 3.09 | Sandwich | Zhao *et al*. [S15] |
| VG/PEDOT | 4.6 | -80 | 1220 | 5.2 | Planar | Hu *et al*. [S16] |
| AEC | 110/100 | -83.5 | 1535 | 0.08 | AEC | Panasonic |
| **3D-CACT** | **12** | **-80.2** | **957** | **3.23** | **Sandwich** | **This work** |

VOGN: vertically oriented graphene nanosheets; ErGO: electrochemically reduced graphene oxide; POG: perpendicularly oriented graphene; NA: not available; X, Y-BWC: carbon membrane from mechanically pressed X,Y-balsa wood; PEDOT:PSS: poly(3,4-ethylenedioxythiophene):poly(styrene sulfonate); HPD-9: sulfuric acid treated PEDOT:PSS film (HP) / PEDOT:PSS film with added DMSO (PD) electrodes, 9 (μL), the mixing solution volume of PEDOT:PSS/DMSO; CPN/p-CNT: continuous PEDOT nano mesh / porous carbon nanotube; G/VACNTs: graphite/vertically aligned carbon nanotubes; SVGA: strictly vertical graphene array; EOG: edge-oriented graphene; 3D-RCT: three-dimensional carbon tube with a rough surface; 3D-TLCT: three-dimensional triple-layer carbon tube; PKHNs: PEDOT: PSS/Ketjenblack holey nanosheets; VG/PEDOT: vertical reduced graphene oxide/PEDOT:PSS. *f*_-45_, the frequency at a phase of -45°.

**Table S2** Comparison of 10 EDLCs based on 3D-CACT electrodes (with area of 1 cm^2^) with the EDLCs in series in terms of ESR, *C'*, *C"*, *X*_C_*,* and phase angle at 120 Hz

| Samples | ESR(Ω) | *C’* at 120 Hz (mF cm^-2^) | *C’’* at 120 Hz (mF cm^-2^) | *X*_C_ at 120 Hz (Ω) | | Phase angle at 120 Hz (°) | |
| --- | --- | --- | --- | --- | --- | --- | --- |
| EDLC 1 | 0.110 | 1.63 | 0.27 | | 0.83 | | -80.4 |
| EDLC 2 | 0.100 | 1.63 | 0.28 | | 0.83 | | -80.3 |
| EDLC 3 | 0.099 | 1.47 | 0.23 | | 0.92 | | -81.2 |
| EDLC 4 | 0.103 | 1.50 | 0.25 | | 0.90 | | -80.7 |
| EDLC 5 | 0.118 | 1.46 | 0.25 | | 0.92 | | -80.4 |
| EDLC 6 | 0.108 | 1.60 | 0.30 | | 0.84 | | -80.1 |
| **6 Devices**  **In series** | **0.618** | **0.27** | **0.04** | | **5.21** | | **-81.0** |
| EDLC 7 | 0.099 | 1.64 | 0.27 | | 0.82 | | -80.7 |
| EDLC 8 | 0.103 | 1.56 | 0.25 | | 0.87 | | -81.0 |
| EDLC 9 | 0.114 | 1.51 | 0.26 | | 0.89 | | -80.1 |
| EDLC 10 | 0.116 | 1.47 | 0.27 | | 0.91 | | -81.2 |
| **10 Devices In series** | **1.019** | **0.16** | **0.028** | | **8.24** | | **-80.6** |

**Supplementary References**

1. T. Natsuki, K. Tantrakarn, M. Endo, Effects of carbon nanotube structures on mechanical properties. Appl. Phys. A **79**, 117-124 (2004). <https://doi.org/10.1007/s00339-003-2492-y>
2. J. R. Miller, R. A. Outlaw, B. C. Holloway, Graphene double-layer capacitor with ac line-filtering performance. Science **329**, 1637-1639 (2010). <https://doi.org/10.1126/science.1194372>
3. K. Sheng, Y. Sun, C. Li, W. Yuan, G. Shi, Ultrahigh-rate supercapacitors based on eletrochemically reduced graphene oxide for ac line-filtering. Sci. Rep. **2**, 247-251 (2012). <https://doi.org/10.1038/srep00247>
4. G. Ren, X. Pan, S. Bayne, Z. Fan, Kilohertz ultrafast electrochemical supercapacitors based on perpendicularly-oriented graphene grown inside of nickel foam. Carbon **71**, 94-101 (2014). <https://doi.org/10.1016/j.carbon.2014.01.017>
5. M. Zhang, W. Wang, L. Tan, M. Eriksson, M. Wu et al., From wood to thin porous carbon membrane: Ancient materials for modern ultrafast electrochemical capacitors in alternating current line filtering. Energy Stor. Mater. **35**, 327-333 (2021). <https://doi.org/10.1016/j.ensm.2020.11.007>
6. M. Zhang, X. Yu, H. Ma, W. Du, L. Qu et al., Robust graphene composite films for multifunctional electrochemical capacitors with an ultrawide range of areal mass loading toward high-rate frequency response and ultrahigh specific capacitance. Energy Environ. Sci. **11**, 559-565 (2018). <https://doi.org/10.1039/c7ee03349d>
7. Z. Li, L. Zhao, X. Zheng, P. Lin, X. Li et al., Continuous pedot:Pss nanomesh film: Towards aqueous ac line filtering capacitor with ultrahigh energy density. Chem. Eng. J. **430**, 133012-133019 (2022). <https://doi.org/10.1016/j.cej.2021.133012>
8. Z. Li, X. Wang, L. Zhao, F. Chi, C. Gao et al., Aqueous hybrid electrochemical capacitors with ultra-high energy density approaching for thousand-volts alternating current line filtering. Nat. Commun. **13**, 6359-6369 (2022). <https://doi.org/10.1038/s41467-022-34082-2>
9. Q. Li, S. Sun, A. D. Smith, P. Lundgren, Y. Fu et al., Compact and low loss electrochemical capacitors using a graphite / carbon nanotube hybrid material for miniaturized systems. J. Power Sources **412**, 374-383 (2019). <https://doi.org/10.1016/j.jpowsour.2018.11.052>
10. S. Xu, Y. Wen, Z. Chen, N. Ji, Z. Zou et al., Vertical graphene arrays as electrodes for ultra-high energy density ac line-filtering capacitors. Angew. Chem. Int. Ed. **60**, 24505-24509 (2021). <https://doi.org/10.1002/anie.202111468>
11. D. Premathilake, R. A. Outlaw, R. A. Quinlan, S. G. Parler, S. M. Butler et al., Fast response, carbon-black-coated, vertically-oriented graphene electric double layer capacitors. J. Electrochem. Soc. **165**, A924-A931 (2018). <https://doi.org/10.1149/2.051805jes>
12. W. Li, S. Azam, G. Dai, Z. Fan, Prussian blue based vertical graphene 3d structures for high frequency electrochemical capacitors. Energy Stor. Mater. **32**, 30-36 (2020). <https://doi.org/10.1016/j.ensm.2020.07.016>
13. F. Han, O. Qian, G. Meng, D. Lin, G. Chen et al., Structurally integrated 3d carbon tube grid-based high-performance filter capacitor. Science **377**, 1004-1007 (2022). <https://doi.org/10.1126/science.abh4380>
14. G. Chen, F. Han, D. Lin, S. Zhang, Q. Pan et al., Three-dimensional multi-layer carbon tube electrodes for ac line-filtering capacitors. Joule **8**, 1080-1091 (2024). <https://doi.org/10.1016/j.joule.2024.01.026>
15. M. Zhao, Y. Qin, X. Wang, L. Wang, Q. Jin et al., Pedot:Pss/ketjenblack holey nanosheets with ultrahigh areal capacitance for khz ac line‐filtering micro‐supercapacitors. Adv. Funct. Mater. 2313495-2313501 (2023). <https://doi.org/10.1002/adfm.202313495>
16. Y. Hu, M. Wu, F. Chi, G. Lai, P. Li et al., Ultralow-resistance electrochemical capacitor for integrable line filtering. Nature **624**, 74-79 (2023). <https://doi.org/10.1038/s41586-023-06712-2>
